# Mechanism of disease and therapeutic rescue of *Dok7* congenital myasthenia

**DOI:** 10.1038/s41586-021-03672-3

**Published:** 2021-06-23

**Authors:** Julien Oury, Wei Zhang, Nadia Leloup, Akiko Koide, Alexis D. Corrado, Gayatri Ketavarapu, Takamitsu Hattori, Shohei Koide, Steven J. Burden

**Affiliations:** 1 Helen L. and Martin S. Kimmel Center for Biology and Medicine at the Skirball Institute of Biomolecular Medicine, NYU Grossman School of Medicine, New York, NY USA; 2 Perlmutter Cancer Center, NYU Langone Health, New York, NY USA; 3 Department of Medicine, NYU Grossman School of Medicine, New York, NY USA; 4 Department of Biochemistry and Molecular Pharmacology, NYU Grossman School of Medicine, New York, NY USA

**Keywords:** Developmental disorders, Neuromuscular disease

## Abstract

Congenital myasthenia (CM) is a devastating neuromuscular disease, and mutations in DOK7, an adaptor protein that is crucial for forming and maintaining neuromuscular synapses, are a major cause of CM^1,2^. The most common disease-causing mutation (*DOK7*^*1124_1127 dup*^) truncates DOK7 and leads to the loss of two tyrosine residues that are phosphorylated and recruit CRK proteins, which are important for anchoring acetylcholine receptors at synapses. Here we describe a mouse model of this common form of CM (*Dok7*^*CM*^ mice) and a mouse with point mutations in the two tyrosine residues (*Dok7*^*2YF*^). We show that *Dok7*^*CM*^ mice had severe deficits in neuromuscular synapse formation that caused neonatal lethality. Unexpectedly, these deficits were due to a severe deficiency in phosphorylation and activation of muscle-specific kinase (MUSK) rather than a deficiency in DOK7 tyrosine phosphorylation. We developed agonist antibodies against MUSK and show that these antibodies restored neuromuscular synapse formation and prevented neonatal lethality and late-onset disease in *Dok7*^*CM*^ mice. These findings identify an unexpected cause for disease and a potential therapy for both *DOK7* CM and other forms of CM caused by mutations in *AGRIN*, *LRP4* or *MUSK*, and illustrate the potential of targeted therapy to rescue congenital lethality.

## Abstract (web summary)

In a mouse model of congenital myasthenia caused by mutations in the *Dok7* gene, agonist antibodies against MUSK restore synaptic function and survival.

## Main

Congenital myasthenia is a group of diseases caused by mutations in genes that are important for the formation, function, and maintenance of neuromuscular synapses^1,2^. Mostly, mutations in these genes are recessive and diminish gene activity, thereby causing synaptic deficits that lead to early onset structural and functional deficits in the neuromuscular synapse, which are responsible for muscle weakness throughout life.

The formation and maintenance of neuromuscular synapses requires the assembly of highly specialized presynaptic and postsynaptic membranes, which involves the coordinated action of several key molecules^3–5^. AGRIN, which is released from motor nerve terminals, binds to the lipoprotein receptor-related protein 4 (LRP4) in muscle, stimulating the formation of a complex between LRP4 and muscle-specific kinase (MUSK), a receptor tyrosine kinase that acts as a master regulator of synaptic differentiation^4–9^. LRP4, once clustered in the postsynaptic membrane as a consequence of MUSK activation, also signals directly back to motor axons to stimulate presynaptic differentiation^10^. Mutations in *AGRIN*, *LRP4* and *MUSK*, as well as in the genes that encode subunits of acetylcholine receptors (AChRs), also cause CM^2,11^.

**Fig. 1:**
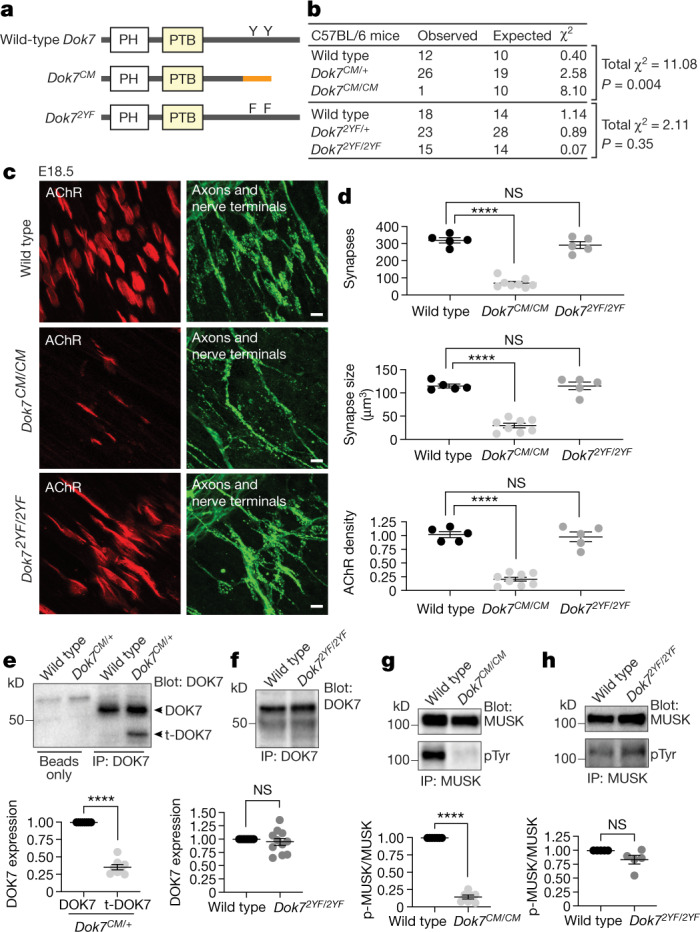
The C-terminal region of DOK7 is essential for synaptic differentiation and to sustain MUSK tyrosine phosphorylation. **a**, *Dok7*^*1124_1127,dup*^ (*Dok7*^*CM*^) leads to a frame-shift and premature termination, including loss of Y396 and Y406. **b**, Expected and observed numbers of progeny, and *χ*^2^ values, from intercrossing *Dok7* heterozygous mutant mice. **c**, **d**, Staining of AChRs (red) and axons and nerve terminals (green) in diaphragm muscles from wild-type and *Dok7* mutant E18.5 mice (**c**). Scale bars, 10 μm. Scatter plots (**d**) show the number of synapses, synaptic size, and density of synaptic AChRs from *n* = 5–8 mice of each genotype. **e**, **f**, Western blot (top) and quantification (bottom) of DOK7 expression in E18.5 mice. *n* = 8 mice in **e**, 11 in **f**. t-DOK7, truncated DOK7; IP, immunoprecipitation. **g**, **h**, Western blot (top) and quantification (bottom) of MUSK tyrosine phosphorylation (pTyr) in E18.5 mice. *n* = 7 mice in **g**, 5 in **h**. Plots show data for individual mice and mean ± s.e.m.; NS, not significant; ****P < 0.00005; two-sided Student’s *t*-test. Source data

Activation of MUSK also depends on the adaptor protein DOK7^12^. Mutations in *Dok7* are responsible for 10–20% of all cases of CM^13–15^. The disease is debilitating—causing weakness in limb, neck and facial muscles—and one-quarter of patients with *DOK7* CM require non-invasive ventilation at some point during their lifetime. Few treatments abate the clinical symptoms^16^. The N-terminal region of DOK7 contains pleckstrin homology (PH) and phosphotyrosine-binding (PTB) domains (Fig. 1a), which function to dimerize DOK7 and bind a phosphorylated tyrosine motif in the MUSK juxtamembrane (JM) region^17^. A failure of DOK7 to bind MUSK leads to a failure of AGRIN to stimulate MUSK phosphorylation^12,18^, demonstrating that DOK7 is essential to stabilize phosphorylation of MUSK, probably by promoting its dimerization^19^. In addition, AGRIN-stimulated MUSK phosphorylation leads to phosphorylation of two tyrosine residues in the C-terminal region of DOK7, which triggers the recruitment of CRK and CRK-L—proteins that participate in the clustering of AChRs^15,20^.

The most common cause of *Dok7* CM is a four-base-pair duplication (residues 1124–1127, TGCC), which leads to a frameshift and premature termination of DOK7^13,21^. Some individuals with *Dok7* CM are homozygous for this mutant allele, whereas others carry this mutant allele in combination with a different mutant allele of *Dok7*. The truncated form of DOK7 retains the PH and PTB domains and binds to the tyrosine-phosphorylated JM region of MUSK^13^, but lacks the two tyrosine residues that are phosphorylated and recruit CRK proteins, suggesting that the loss of these tyrosine residues is responsible for the synaptic deficits in this common form of *Dok7* CM^2,15,20^.

## Synapse formation requires DOK7 C-terminal region

To study how loss of the C-terminal region of DOK7 leads to defects in the structure and function of neuromuscular synapses, we generated a mouse model of the most common form of *Dok7* CM (*Dok7*^*1124_1127 dup*^; referred to as *Dok7*^*CM*^ mice) and a second mouse mutant (*Dok7*^*Y396F,Y406F*^; referred to as *Dok7*^*2YF*^ mice), in which the two tyrosine residues in the C-terminal region are mutated to phenylalanine (Fig. 1a).

**Extended Data Fig. 1:**
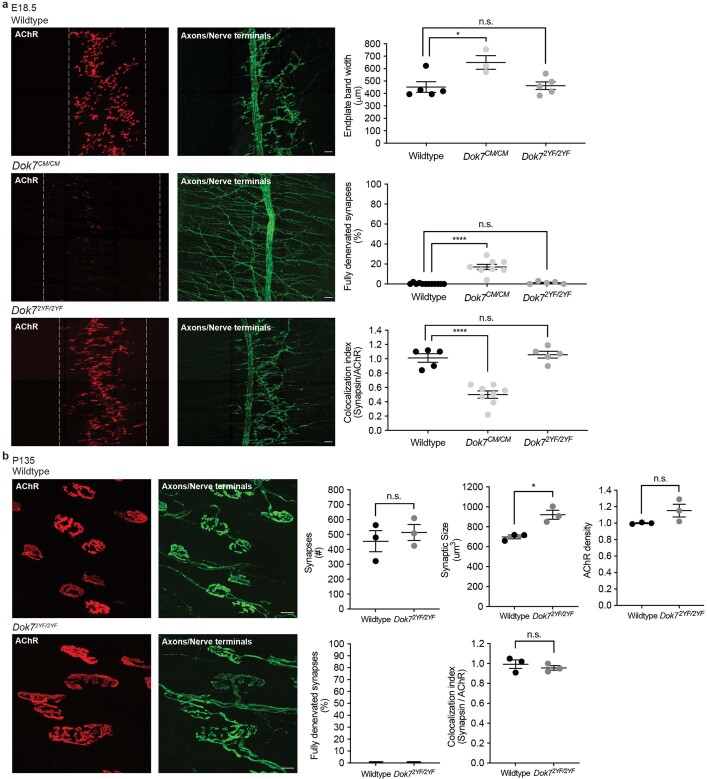
Characterization of neuromuscular synapses in *Dok7* mutant mice. **a**, **b**, Left, diaphragm muscles from E18.5 (**a**) and P135 (**b**) wild-type and *Dok7* mutant mice were stained with Alexa 488–anti-BGT to label AChRs (red) and antibodies against neurofilament and synapsin to label motor axons and nerve terminals (green). Scale bars, 50 μm (**a**), 10 μm (**b**). **a**, Right, endplate width, denervation and co-localization of synapses in wild-type, *Dok7*^*CM/CM*^ and *Dok7*^*2YF/2YF*^ mice. The width of the endplate band (dashed lines) was increased by 45% in *Dok7*^*CM/CM*^ mice but was normal in *Dok7*^*2YF/2YF*^ mice. In *Dok7*^*CM/CM*^ mice, 17% of AChR clusters were completely unopposed by nerve terminals, indicating denervated myofibres. Many synapses in *Dok7*^*CM/CM*^ mice were partially innervated, as nearly half of the AChR-rich area at synapses was not juxtaposed by nerve terminals. **b**, Right, in *Dok7*^*2YF/2YF*^ mice, synapses mature from a plaque-like to a complex, pretzel-like shape, characteristic of mature mouse neuromuscular synapses. Synapses in *Dok7*^*2YF/2YF*^ mice, however, often appeared elongated. The number of synapses and the density of synaptic AChRs were similar in wild-type and *Dok7*^*2YF/2YF*^ mice. Synaptic size was increased by 20% in *Dok7*^*2YF/2YF*^ mice when compared with wild-type mice. Data shown as mean ± s.e.m. from 3 mice (>50 synapses per mouse). n.s., not significant; *P < 0.05, ****P < 0.00005; two-sided Student’s *t*-test. Source data

Homozygous *Dok7*^*CM*^ mice were present at the expected numbers at embryonic day 18.5 (E18.5), but were rarely found alive a day later, at birth, when neuromuscular synapses are essential for respiration and survival (Fig. 1b). We stained diaphragm muscles from E18.5 embryos with probes that allowed us to visualize presynaptic and postsynaptic differentiation and found fivefold fewer synapses in *Dok7*^*CM*^ mice than in wild-type mice (Fig. 1c, d). Moreover, the synapses that did form were immature, as both synaptic size and the density of synaptic AChRs were reduced fivefold (Fig. 1c, d, Extended Data Fig. 1a). By contrast, homozygous *Dok7*^*2YF*^ mice were born at the expected frequency (Fig. 1b) and thrived as fertile adult mice. Moreover, their neuromuscular synapses appeared largely normal (Fig. 1c, d, Extended Data Fig. 1b). Thus, unexpectedly, loss of the two tyrosine residues in the C-terminal region of DOK7 is not the cause of the lethality and severe synaptic deficits in *Dok7*^*CM*^ mice.

## *Dok7*^*CM*^ lowers DOK7 levels and MUSK phosphorylation

**Extended Data Fig. 2:**
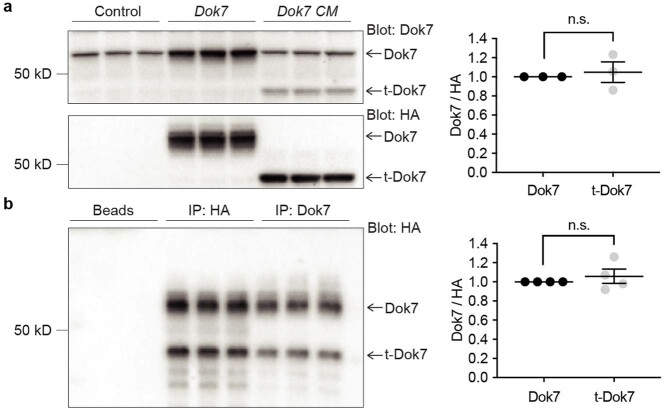
Wild-type and truncated DOK7 are detected with similar efficiency by antibodies against the PH and PTB domains in DOK7. **a**, HEK 293 cells were transiently transfected with a plasmid expressing either HA-tagged DOK7 or HA-tagged truncated DOK7 encoded by *Dok7*^*1124_1127 TGCC dup*^. Proteins in cell lysates (triplicates) were separated by SDS–PAGE, and western blots were probed with either a rabbit antibody against the PTB domain in DOK7 or a monoclonal antibody against HA (left). We measured the grey levels of the bands for wild-type and truncated DOK7 proteins and normalized the level detected by western blotting with the rabbit antibody against DOK7 with the level detected by western blotting with the antibody against HA. The ratio for wild-type DOK7 was equivalent to the ratio for truncated DOK7 (left), indicating that the rabbit antibody against DOK7 detected wild-type and truncated DOK7 proteins with similar efficiency by western blotting. **b**, Wild-type and truncated DOK7 were immunoprecipitated with similar efficiency by a goat antibody against the PTB domain in DOK7. HEK 293 cells were transiently co-transfected with plasmids expressing HA-tagged DOK7 and HA-tagged truncated DOK7 encoded by *Dok7*^*1124_1127 TGCC dup*^. DOK7 proteins were immunoprecipitated from cell lysates (triplicates) with either a monoclonal antibody against HA or a goat antibody against the PTB domain in DOK7, and western blots were probed with the monoclonal antibody against HA (left). We measured the grey levels, subtracted the level for the background band in the control, non-transfected samples, and normalized the value for each protein immunoprecipitated with the goat-antibody against DOK7 to the value for the same protein immunoprecipitated with the antibody against HA. This ratio was equivalent for wild-type and truncated DOK7 proteins, indicating that the goat antibody against DOK7 immunoprecipitated wild-type and truncated proteins with similar efficiency (right). Plots show individual values from three and four experiments for **a** and **b**, respectively, and the mean ± s.e.m. (n.s., not significant); two-sided Student’s *t*-test. Source data

**Extended Data Fig. 3:**
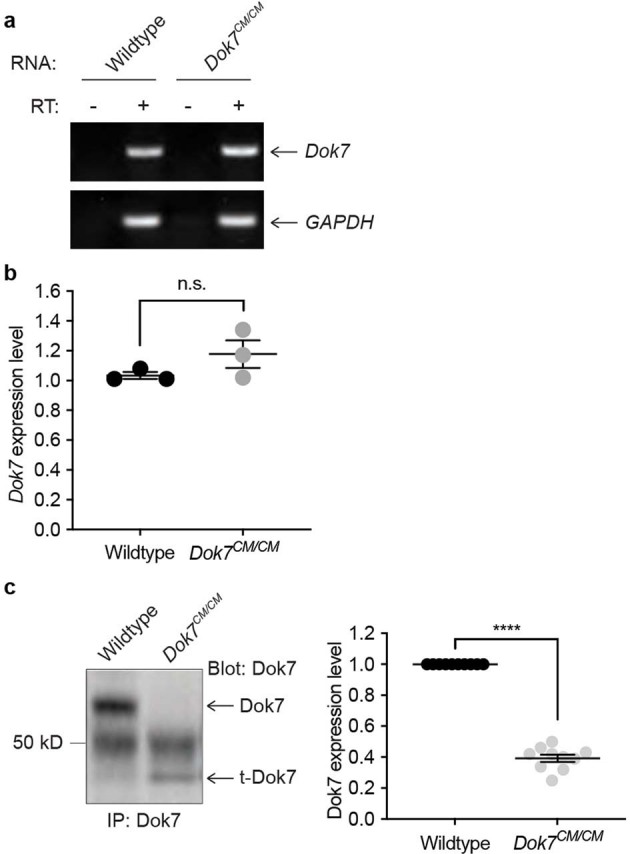
*Dok7* RNA expression is normal in *Dok7*^*CM/CM*^ mice. **a**, RT–PCR amplification of *Dok7* RNA shows that *Dok7* mRNA levels are similar in muscle from E18.5 wild-type and *Dok7*^*CM/CM*^ mice. *GAPDH* was used as a loading control. **b**, *Dok7* mRNA levels were quantified by qPCR, which showed that *Dok7* mRNA levels are normal in *Dok7*^*CM/CM*^ mice (*n* = 3 mice). **c**, DOK7 was immunoprecipitated from muscles of E18.5 wild-type and *Dok7*^*CM/CM*^ mice, and the blots were probed with antibodies against DOK7 (left). Truncated DOK7, encoded by *Dok7*^*CM/CM*^, migrates at the predicted size, but is expressed at threefold lower levels than wild-type DOK7 (right; *n* = 10 mice). Data shown as individual data points and mean ± s.e.m.; n.s., not significant; ****P < 0.00005; two-sided Student’s *t*-test. Source data

**Extended Data Fig. 4:**
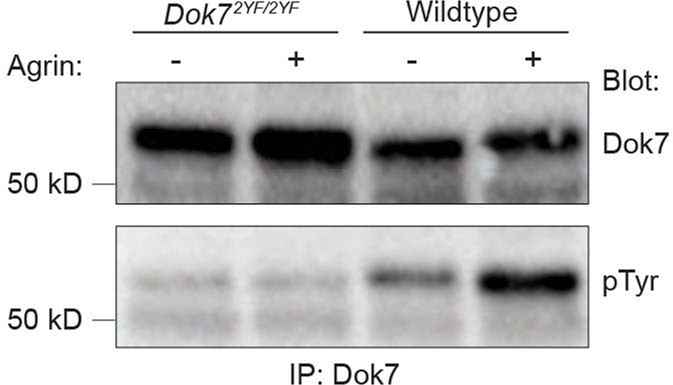
Y396 and Y406 are the main, if not sole, tyrosine residues in DOK7 that are phosphorylated by AGRIN stimulation. We generated muscle cell lines from wild-type and *Dok7*^*2YF/2YF*^ mice and treated the cultured myotubes with AGRIN for 30 min. MUSK was immunoprecipitated, and western blots were probed with antibodies against MUSK or phosphotyrosine (pTyr). AGRIN stimulates DOK7 tyrosine phosphorylation in wild-type but not *Dok7*^*2YF/2YF*^ myotubes (data from two experiments).

To determine how loss of the DOK7 C-terminal region caused the synaptic defects, we measured expression of *Dok7* mRNA and truncated DOK7 protein in *Dok7*^*CM*^ mice using antibodies against the DOK7 PTB domain that detected the truncated and wild-type proteins equally (Extended Data Fig. 2). *Dok7* mRNA levels were normal in muscle from *Dok7*^*CM*^ mice, whereas the truncated DOK7 protein was expressed at threefold lower levels than the wild-type DOK7 protein (Fig. 1e, Extended Data Fig. 3). By contrast, DOK7(2YF) was expressed at normal levels (Fig. 1f) and, as expected^15,20^, was not tyrosine phosphorylated (Extended Data Fig. 4).

Because DOK7 functions as a dimer to dimerize MUSK, thereby stabilizing MUSK tyrosine phosphorylation^19^, we determined whether MUSK tyrosine phosphorylation was diminished in *Dok7*^*CM*^ mice. MUSK phosphorylation was reduced sevenfold in *Dok7*^*CM*^ mice but was normal in *Dok7*^*2YF*^ mice (Fig. 1g, h).

## CRK proteins are recruited directly to MUSK

**Fig. 2:**
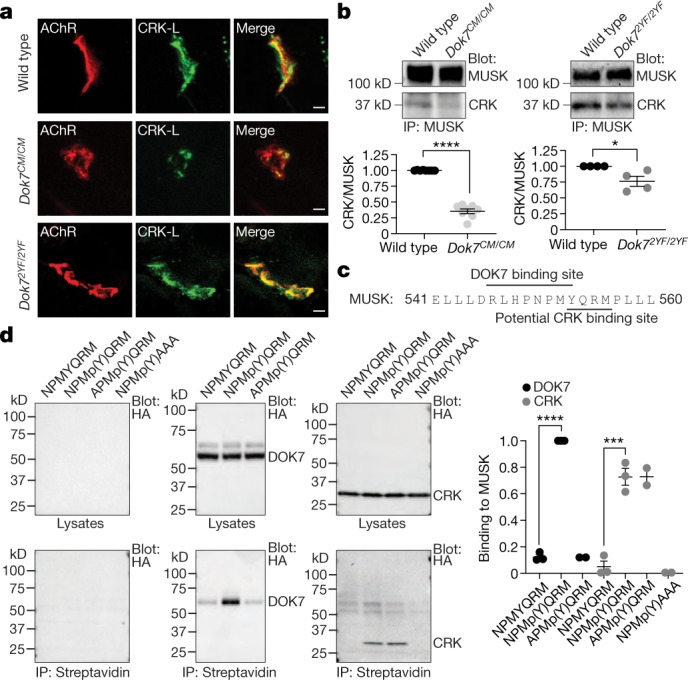
Recruitment of CRK to the synapse and to the MUSK–DOK7 complex is impaired in *Dok7*^*CM/CM*^ mice. **a**, Staining for CRK-L (green) and AChRs (red) in muscle sections from E18.5 mice. Scale bars, 5 μm. Representative images from three experiments. **b**, Top, co-immunoprecipitation of CRK with MUSK from muscles of E18.5 mice. Bottom, CRK levels normalized to MUSK levels for mice of each genotype (*n* = 8 for *Dok7*^*CM/CM*^ and *n* = 4 for *Dok7*^*2YF/2YF*^mice). **c**, Amino acid sequence of the MUSK JM region showing binding site for DOK7 and potential binding site for CRK. **d**, Left, affinity capture of DOK7 and CRK-I with phosphorylated and non-phosphorylated peptides detected by immunoblotting. The peptide sequences are shown. Right, quantification. Plots show individual data and mean ± s.e.m.; *P < 0.05, ***P < 0.0005, ****P < 0.00005; two-sided Student’s *t*-test. Source data

We anticipated that recruitment of CRK proteins to the synapse would be absent or severely reduced in both *Dok7*^*CM*^ and *Dok7*^*2YF*^ mutant mice. Indeed, CRK recruitment to the synapse and to the MUSK complex was substantially diminished (2.8-fold) in *Dok7*^*CM*^ mice (Fig. 2a, b), but to our surprise was only modestly reduced (by 28%) in *Dok7*^*2YF*^ mice (Fig. 2a, b). These findings suggest that CRK is recruited to a tyrosine phosphorylated synaptic protein(s) in addition to DOK7.

The three activation loop tyrosines and Y553 in MUSK become phosphorylated after stimulation by AGRIN^12,18,22,23^. We found that Y553 in the MUSK JM region is within not only a PTB-binding site that recruits DOK7, but also a potential SH2-binding motif for CRK proteins (Fig. 2c). Both CRKI and DOK7 bound the MUSK JM site in a phosphorylation-dependent manner (Fig. 2d). Mutation of amino acids that compose the SH2-binding motif, but not the PTB-binding site, impaired binding of CRKI (Fig. 2d). Thus, CRK can bind not only to the phosphorylated C-terminal region of DOK7 but also directly to the tyrosine-phosphorylated JM region of MUSK. This redundancy for recruiting CRK to the synapse is likely to explain the near-normal association of CRK with the MUSK complex in *Dok7*^*2YF*^ mice and the difference in the phenotypes of *Dok7*^*CM*^ and *Dok7*^*2YF*^ mice.

## Agonist antibodies against MUSK

If diminished MUSK phosphorylation caused disease in *Dok7* CM, we reasoned that stimulation of MUSK might rescue the synaptic defects and overcome lethality. We explored this idea by generating agonist antibodies targeting MUSK and treating *Dok7*^*CM*^ mice with the agonist antibodies.

We screened a phage-display library for antibodies that bound the Fz-like domain in the extracellular region of mouse and human MUSK. We targeted the Fz-like domain because this domain is not essential for MUSK function and antibodies against the Fz-like domain cause no obvious harm in mice^24,25^.

**Fig. 3:**
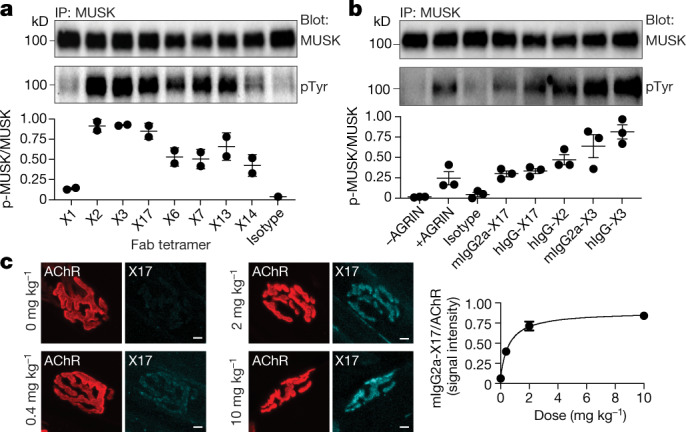
Antibodies against MUSK Fz domain stimulate MUSK phosphorylation in cultured myotubes and bind MUSK in vivo. **a**, MUSK phosphorylation, normalized to MUSK expression, in C2 myotubes treated for 30 min with biotinylated Fabs each tetramerized with streptavidin. Top, western blot; bottom, quantification. **b**, MUSK phosphorylation in C2 myotubes treated with 0.5 nM AGRIN or 10 nM antibodies, with either mouse IgG2a or human IgG1 Fc regions. Top, western blot; bottom, quantification. **c**, Left, staining for AChRs (red) and human IgG (cyan) in diaphragm muscles of P30 wild-type mice two days after intraperitoneal injection of antibody X17. Right, X17 signal intensities normalized to AChR plotted against antibody dose (*n* = 3 mice per concentration). Plots show mean ± s.e.m. (with individual data points in **a**, **b**). Source data

**Extended Data Fig. 5:**
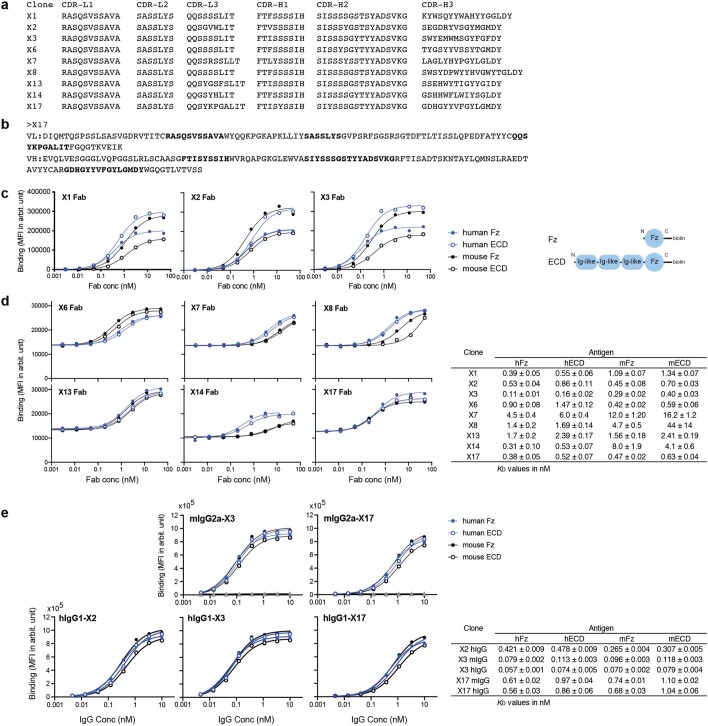
MUSK antibody clones. **a**, Amino acid sequences of the complementarity-determining regions (CDRs) of antibodies against MUSK developed in this study. CDR definitions are based on Wu and Kabat^38^, except that CDR-H1 includes four additional residues at the N terminus to show diversified positions. **b**, Amino acid sequences of the VL and VH domains of clone X17. **c**, **d**, Binding titration of antibodies against MUSK in the Fab format to immobilized hFz, hECD, mFz and mECD, as tested using a bead-based binding assay. Curves show the best fit of the 1:1 binding model. The table lists apparent *K*_D_ values (mean ± s.d., *n* = 3). The datasets in **c** and **d** were obtained different instruments, which resulted in different signal ranges. **e**, Binding titration of antibodies against MUSK in the IgG format, as in **c**. Source data

We identified high-affinity antibodies that bound the Fz-like domain in human and mouse MUSK and stimulated MUSK phosphorylation independent of AGRIN (Fig. 3a, b, Extended Data Fig. 5), thereby overcoming the shortcomings of previously described agonist antibodies that recognized mouse but not human MUSK^25^.

**Extended Data Fig. 6:**
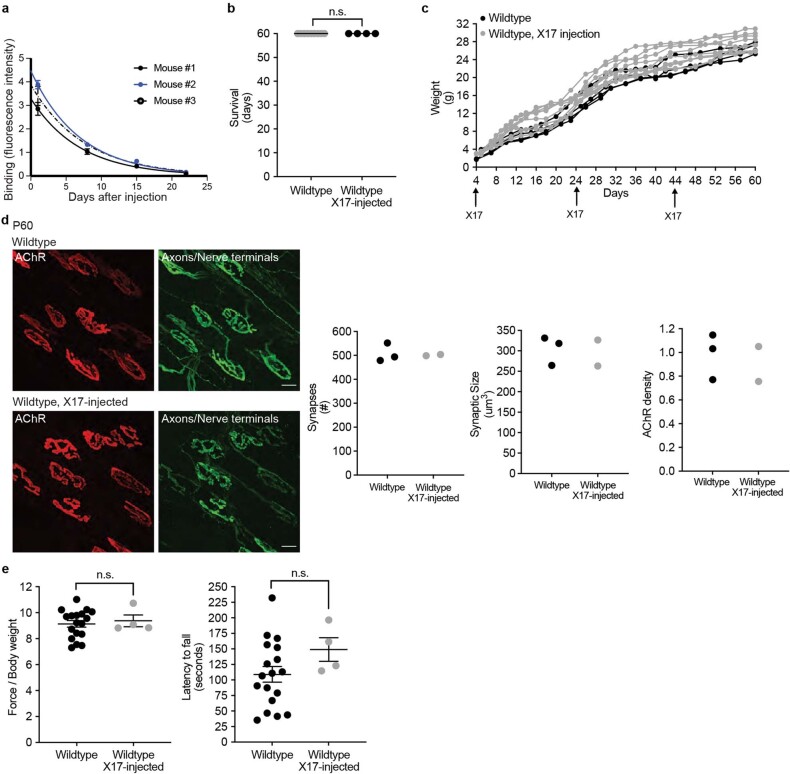
Chronic injection of MUSK agonist antibody X17 in wild-type mice has no effect on the organization of neuromuscular synapses, weight gain or motor behaviour. **a**, Blood half-life measurements of X17-mIgG2a-LALAPG. Nonlinear least-squares fitting of the median fluorescence intensities with a single exponential curve for three mice is shown. The half-life was determined to be 4.9 ± 0.2 days. **b**, Wild-type mice on a C57BL/6-CBA mixed background, injected at P4, P24 and P44 with X17 (*n* = 4), survived until P60, when the experiment was ended. The scatter plot shows the survival time for nine non-injected wild-type mice and four wild-type mice injected with X17 with mean ± s.e.m. (n.s., not significant). **c**, Wild-type mice injected with X17 (*n* = 4) gained weight like uninjected wild-type mice (*n* = 9). **d**, Left, diaphragm muscles from P60 wild-type mice and wild-type mice injected with X17 were stained with Alexa 488–anti-BGT to label AChRs (red) and antibodies against neurofilament and synapsin to label motor axons and nerve terminals (green). In wild-type mice treated with X17, synapses matured from a simple, plaque-like shape to a complex, pretzel-like shape, characteristic of mature mouse neuromuscular synapses. Scale bar, 10 μm. Right, injection of X17 in wild-type mice had no effect on synapse number, size or AChR density. We analysed more than 50 synapses per diaphragm muscle from two mice in each category. **e**, Motor performance of wild-type mice injected with X17, as assessed by grip strength and the latency to fall from a rotating rotarod, was similar to that of non-injected wild-type mice. The scatter plots show the values for 18 wild-type mice and 4 wild-type mice injected with X17 and the mean ± s.e.m; two-sided Student’s *t*-test. Source data

We injected the MUSK agonist antibody X17, in a mouse IgG2a format with mutations that reduce Fc domain effector function^26^, interperitoneally into wild-type mice and found that 10 mg kg^−1^ of X17 had a half-life of 5 days in blood and that it saturated synaptic MUSK (Fig. 3c, Extended Data Fig. 6a). Chronic injection of X17 (10 mg kg^−1^ at postnatal day 4 (P4), P24 and P44) in wild-type mice over two months had no effect on the organization of neuromuscular synapses, weight gain or motor behaviour (Extended Data Fig. 6).

## X17 rescues synapse formation and lethality

**Fig. 4:**
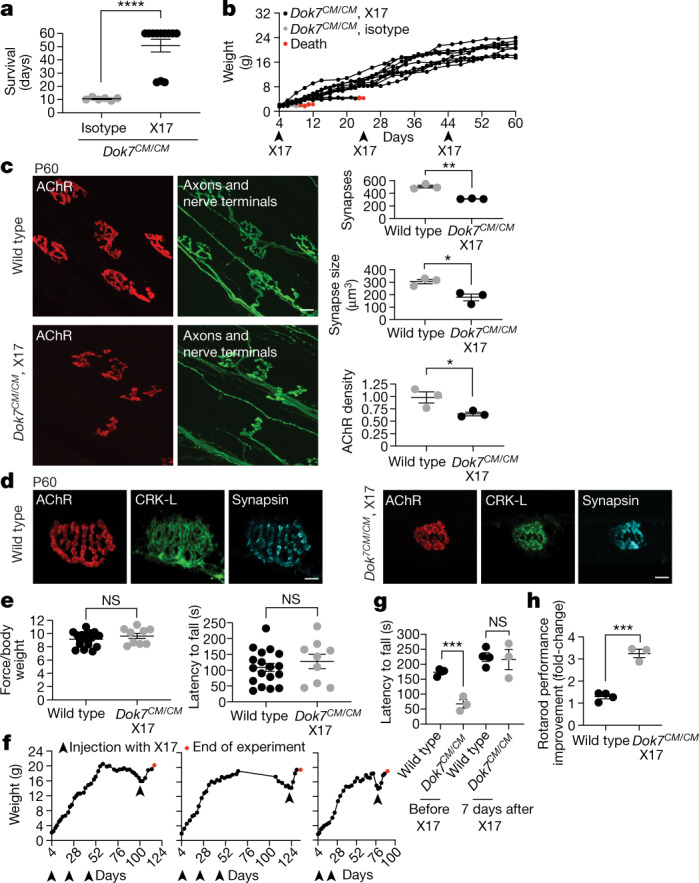
An agonist antibody against MUSK restores synapse development and rescues lethality in young *Dok7*^*CM*^ mice and reverses disease relapse in adult *Dok7*^*CM*^ mice. **a**, *Dok7*^*CM/CM*^ mice on a C57BL/6-CBA mixed background (*n* = 12) were injected with X17 at P4, P24 and P44, and the experiment was ended when the mice were at P60. Mice (*n* = 6) injected with the isotype control died within two weeks of birth. **b**, X17 restored weight gain in *Dok7*^*CM/CM*^ mice. **c**, Left, diaphragm muscles from P60 mice stained for AChRs (red) and neurofilament and synapsin to label motor axons and nerve terminals (green). Scale bar, 10 μm. Right, quantification (n = 3 mice, >50 synapses per mouse). **d**, Staining for AChRs (red), CRK-L (green) and synapsin (cyan) in myofibres isolated from muscles of wild-type mice (left) and *Dok7*^*CM/CM*^ mice injected with X17 (right). Representative images of ten synapses per mouse from three mice. Scale bars, 5 μm. **e**, Grip strength and latency to fall from a rotating rotarod of *Dok7*^*CM/CM*^ mice treated with X17 (*n* = 9), compared with wild-type mice (*n* = 18). **f**, Weight changes in *Dok7*^*CM/CM*^ mice treated either with mIgG2a-X17 at P4, P24 and P44 or with hIgG1-X17 at P4 and P18, with antibody treatment then discontinued for 2–3 months. When *Dok7*^*CM/CM*^ mice began to lose weight and showed motor deficits, they were re-injected with hIgG1-X17 and their weights monitored. Red dots indicate death at the end of the experiment. **g**, Latency to fall for four wild-type and three *Dok7*^*CM/CM*^ mice. **h**, Change in rotarod performance one week after X17 treatment of *Dok7*^*CM/CM*^ mice (fold-change from performance before X17), compared with that of wild-type mice not injected with X17. All plots except **b**, **f** show individual data points and mean ± s.e.m.; NS, not significant; *P < 0.05, **P < 0.005, ***P < 0.0005, ****P < 0.00005; two-sided Student’s *t*-test. Source data

**Extended Data Fig. 7:**
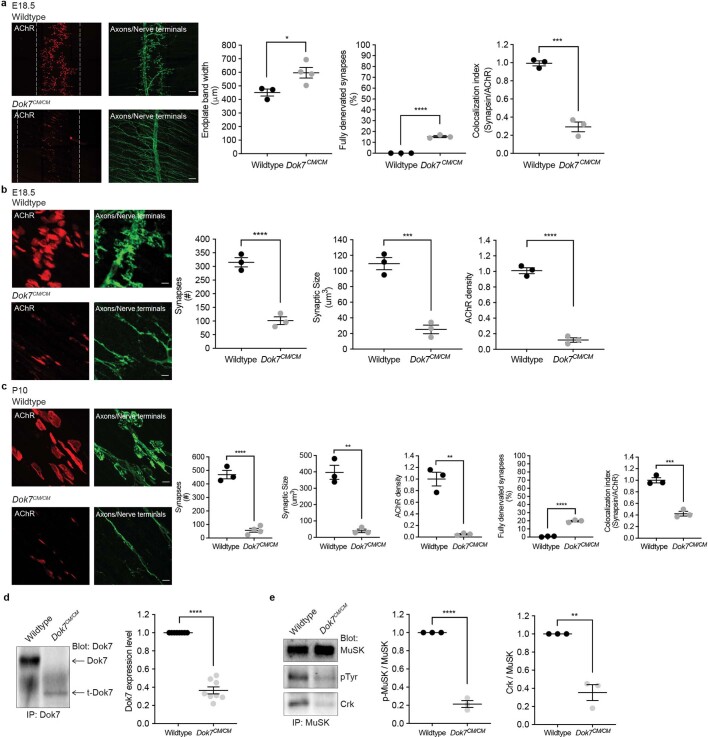
The C-terminal region of DOK7 is essential for complete differentiation and maturation of the neuromuscular synapse in *Dok7*^*CM/CM*^ mice on a mixed genetic background. **a**–**c**, Left, diaphragm muscles from wild-type and *Dok7*^*CM/CM*^ mice on a C57BL/6-CBA mixed background at E18.5 and P10 were stained with Alexa 488–anti-BGT to label AChRs (red) and antibodies against neurofilament and synapsin to label motor axons and nerve terminals (green). Scale bars, 50 μm (**a**), 10 μm (**b**, **c**). **a**, Right, at E18.5, the endplate band (dashed white lines on left) is 30% wider in *Dok7*^*CM/CM*^ than wild-type mice. Moreover, nerve terminals were absent from 15% of AChR clusters and the colocalization index (synapsin/AChR) was reduced 3.5-fold in *Dok7*^*CM/CM*^ mice. *n* = 3 mice. **b**, Right, the number of synapses, synaptic size and density of synaptic AChRs were reduced 3.2-, 4.5- and 8-fold, respectively, in E18.5 *Dok7*^*CM/CM*^ mice. *n* = 3 mice (>50 synapses per mouse). **c**, Right, at P10, the number of synapses, synaptic size and density of synaptic AChRs were reduced over tenfold in *Dok7*^*CM/CM*^ mice. In addition, nerve terminals were absent from 20% of the AChR clusters in *Dok7*^*CM/CM*^ mice. *n* = 3 mice (>50 synapses per mouse). **d**, DOK7 was immunoprecipitated from muscles of E18.5 wild-type and *Dok7*^*CM/CM*^ mice, and the blots were probed with antibodies against DOK7 (left). Truncated DOK7, encoded by *Dok7*^*CM/CM*^, migrated at the predicted size, but was expressed at threefold lower levels than wild-type DOK7 (right). Because DOK7 expression and MUSK phosphorylation were diminished to the same extent in the C57BL/6-CBA mixed breed and C57BL/6 inbred mice, other factors presumably led to increased survival in the mixed genetic background. *n* = 8 mice per genotype. **e**, MUSK was immunoprecipitated from muscles of E18.5 wild-type and *Dok7*^*CM/CM*^ mice, and the blots were probed with antibodies against MUSK, phosphotyrosine, and CRK (left). The levels of phosphotyrosine and CRK that co-isolated with the MUSK complex were normalized to MUSK expression (right). CRK association with the MUSK complex was 2.8-fold lower in *Dok7*^*CM/CM*^ mice than wild-type mice. MUSK tyrosine phosphorylation was fivefold lower in *Dok7*^*CM/CM*^ mice than wild-type mice. *n* = 3 mice per genotype. Scatter plots show individual data points and mean ± s.e.m.; *P < 0.05, **P < 0.005, ***P < 0.0005, ****P < 0.00005; two-sided Student’s *t*-test. Source data

**Extended Data Fig. 8:**
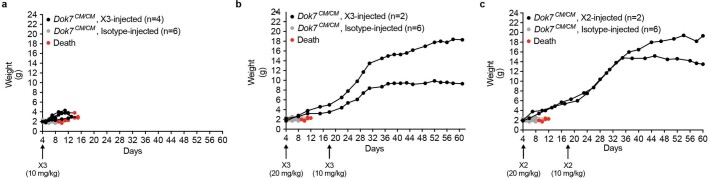
Antibodies X2 and X3, like X17, rescue *Dok7*^*CM/CM*^ mice from early lethality. **a**, *Dok7*^*CM/CM*^ mice on a C57BL/6-CBA mixed background were injected at P4 with 10 mg kg^−1^ mIgG2a-X3. At this dose, X3 failed to rescue the mice from lethality. **b**, By contrast, dosing with 20 mg kg^−1^ mIgG2a-X3 at P4 rescued the mice from early lethality. These mice were subsequently injected with 10 mg kg^−1^ mIgG2a-X3 at P18, which led to survival until P60, when the experiment was ended. **c**, Injecting *Dok7*^*CM/CM*^ mice with 20 mg kg^−1^ hIgG1-X2 at P4 likewise rescued *Dok7*^*CM/CM*^ mice from early lethality; subsequent injection of 10 mg kg^−1^ hIgG1-X2 at P18 led to survival of *Dok7*^*CM/CM*^ mice until P60, when the experiment was ended. Source data

Although *Dok7*^*CM*^ mice on a C57BL/6 background died at birth, we found that *Dok7*^*CM*^ mice on a mixed genetic background survived for one to two weeks after birth (Extended Data Table 1), which facilitated experiments to study the therapeutic efficacy of X17. We injected these *Dok7*^*CM*^ mice with 10 mg kg^−1^ of X17 or an isotype-matched negative control antibody at P4, when they showed signs of disease (they were runted and had synaptic deficits) (Extended Data Fig. 7). *Dok7*^*CM*^ mice injected with the control antibody continued to lose weight and died within a week (Fig. 4a, b). Injection of X17 reversed the weight loss and rescued the *Dok7*^*CM*^ mice from this early lethality (Fig. 4a, b). Over the next few weeks, the weight gain was continuous in nine of the twelve *Dok7*^*CM*^ mice injected with X17; the weight gain slowed in three of the X17-injected mice, and they died at P23–P24. Another antibody, X3, rescued *Dok7*^*CM*^ mice from early postnatal lethality when injected at 20 mg kg^−1^ but not at 10 mg kg^−1^ (Extended Data Fig. 8), suggesting that a higher initial dose of a MUSK agonist antibody may be more effective during early postnatal development, when synapses are undergoing critical steps in maturation.

We investigated whether chronic dosing could lead to long-term survival. Repeated injections of X17 in the nine surviving *Dok7*^*CM*^ mice at P24 and P44 led to survival of these mice for at least two months (Fig. 4a, b), at which point we assessed their motor performance and the structure of their neuromuscular synapses. X17 rescued synapse formation and maturation, as the neuromuscular synapses of these mice had developed the complex pretzel-like shape characteristic of fully mature mouse neuromuscular synapses (Fig. 4c). Moreover, X17 rescued the recruitment of CRK proteins to the neuromuscular synapse (Fig. 4d).

Antibody X17 rescued the motor function of *Dok7*^*CM*^ mice, as assessed by grip strength and rotarod assays (Fig. 4e). Moreover, *Dok7*^*CM*^ mice injected with X17 were fertile and produced offspring at the expected frequency. Together, these findings indicate that reduced MUSK tyrosine phosphorylation is central to disease in *Dok7*^*CM*^ mice. Even if the C-terminal region of DOK7 has an additional role in synapse formation, this function can be overridden by stimulating MUSK.

## Therapeutic reversal in adult *Dok7*^*CM*^ mice

We next sought to determine whether X17 could reverse neuromuscular deficits that develop during adulthood, a question particularly relevant to developing a human therapy as *DOK7* CM in humans would probably be treated during adult life. We treated *Dok7*^*CM*^ mice with X17 either at P4, P24 and P44 or at P4 and P18 but then discontinued antibody treatment. Both groups of *Dok7*^*CM*^ mice continued to maintain their weight and mobility for 2–3 months (Fig. 4f), indicating that the effects of the antibody lasted for longer than its lifetime in the blood. However, mice ultimately began to lose weight and display motor deficits (Fig. 4f, g, Supplementary Video 1). When the mice were losing weight at a rate of about 0.4 g per day, we injected X17 once again and monitored their weight and mobility. Two days after the resumption of X17 treatment, the *Dok7*^*CM*^ mice began to regain weight (by about 0.4 g per day over the next week) (Fig. 4f). Within one week of reinitiating antibody treatment, the motor performance of the *Dok7*^*CM*^ mice had been restored (Fig. 4g, Supplementary Video 1). The mice continued to gain weight and their motor performance continued to improve for at least one additional week after antibody treatment, when the experiment was ended (Fig. 4f, g).

## Discussion

Stimulation of MUSK with an agonist antibody rescued synapse formation and motor function, prevented lethality and allowed *Dok7*^*CM*^ mice to thrive postnatally as fertile adults. Moreover, the motor deficits that developed in adult *Dok7*^*CM*^ mice after withdrawal of antibody treatment were readily reversed by reinitiating antibody treatment. Thus this therapeutic strategy, which avoids the complex requirements for gene therapy^27^, might be beneficial for humans with *DOK7* CM or other neuromuscular diseases.

Most previous studies of DOK7 have relied upon analysis of transfected muscle and non-muscle cells that overexpress DOK7^12,15,20^. In this context, which bypasses the normal requirement for AGRIN and LRP4 to stimulate MUSK, the in vivo consequences of *Dok7* mutations might have been masked by the overexpression of DOK7.

Inbred C57BL/6 mice containing the *Dok7*^*CM*^ mutation showed more severe functional deficits than humans with the same mutation. The mutant phenotype was less severe in mice with a mixed genetic background, as outbred mice survived for up to three weeks postnatally, whereas inbred mutant mice died at birth. Modifiers in the hybrid strains may lessen disease severity, or C57BL/6 mice may contain genes that worsen the phenotype. In either case, the modestly prolonged lifespan of *Dok7*^*CM*^ mice on the mixed background offers a mouse model that presents a longer temporal window in which to assess therapeutic approaches.

These experiments demonstrate full rescue from congenital lethality by targeted therapy. Our findings point to an unforeseen therapeutic approach, as this strategy does not directly target the mutant protein but rather targets a wild-type protein that has diminished activity caused by the mutation of an upstream gene, in this case *DOK7*. Epistatic rescue in this way could also provide therapy for CM caused by mutations in *AGRIN*, *LRP4* or *MUSK*, in addition to *DOK7*, as well as for other neuromuscular diseases. Moreover, this strategy has the potential for widespread use to treat genetic disorders in humans for which the disease mechanism is understood and suitable targets have been identified.

## Methods

### Mice

To generate *Dok7*^*CM*^ mice, we microinjected in vitro-transcribed single guide RNA (sgRNA; 5′ CTGCTCAGTCTGCCCCC 3′, 5 ng/μl) and in vitro-transcribed Cas9 RNA (10 ng/μl), together with a DNA repair template (5′ ATGCCGGCAATCTGGACGTCTGGCGGGCCGGTGAGGAATTCGGTTCTCTGCTCAGTCTGCCTGCCCCCTGGAGCCAGCGCACCTGAGCCCAGACTGTGTGCCTGCCCACCTGGGGCGGCCGAGTA 3′, 10 ng/μl) containing the TGCC duplication, into the pronuclei of C57BL/6 mouse zygotes^28^. We analysed 14 mice that were born from injected zygotes by sequencing tail DNA (primer: 5′ GCAGTTACAGGAGGTTGG 3′). One mouse carried a *Dok7* allele with the desired TGCC duplication. We crossed the founder mouse with wild-type C57BL/6 mice to generate the *Dok7*^*CM*^ line. DNA sequencing confirmed the sequence of the *Dok7* mutation. Mice were subsequently genotyped using primers (forward: 5′ GCGGCCTCGGCAGTTACAG 3′; reverse: 5′ GCTTTACCTTGAGTCCGCCACAGA 3′). We analysed five genomic loci that scored the highest probability for off-target recognition (http://crispr.mit.edu). We found no evidence for mutations in these genes (Extended Data Table 2).

An earlier study described a similar mouse model, generated using classic embryonic stem cell gene targeting, for this common form of *DOK7* CM^27^. Although the lethality of these mutant mice could be rescued by an adenoviral-associated vector expressing wild-type DOK7, establishing a gene therapy approach to treat *DOK7* CM^27^, this study did not examine the cause of disease in the *Dok7*^*1124_1127,dup*^ mouse model.

To generate *Dok7*^*2YF*^ mice, we injected an sgRNA (5′ TTCGAGGTGTGTCATAG 3′, 15 ng/μl) and in vitro-transcribed Cas9 RNA (30 ng/μl), together with the DNA repair template (5′ ATGCCGGCAGCAACCTGGACGTGTGGCGGGCCGGTGAGGAATTCGGTTCTCTGCTCAGTCTGCCTGCCCCCTGGAGCCAGCGCACCTGAGCCCAGACTGTGTGCCTGCCCACCTGGGGCGGCCGAGTA 3′, 30 ng/μl) to convert tyrosine 396 and tyrosine 406 to phenylalanine, into the cytoplasm of C57BL/6 mouse zygotes. We analysed 33 mice that were born from injected zygotes by sequencing tail DNA (primer: 5′ TGGCATTGCCACAGGCAG 3′). One mouse carried a *Dok7* allele with the desired tyrosine-to-phenylalanine substitutions. We crossed the founder mouse with wild-type C57BL/6 mice to generate the *Dok7*^*2YF*^ line. DNA sequencing from these lines confirmed the sequence of the *Dok7* mutation. Mice were housed and maintained according to Institutional Animal Use and Care Committee (IACUC) guidelines.

### Growth of cultured cells

C2C12 mouse muscle cells, purchased from and authenticated by ATCC (Cat CRL-1772), were grown at 37 °C in growth medium (GM): Dulbecco’s modified Eagle’s medium (DMEM) containing 4.5 g/l glucose, l-glutamine and sodium pyruvate (Corning cellgro), supplemented with 10% fetal bovine serum (FBS; GemCell). Myoblast fusion and myotube differentiation were induced when myoblasts were 70% confluent by switching to differentiation medium (DM): DMEM with 4.5 g/l glucose and 1 mM l-glutamine, supplemented with 2% heat-inactivated horse serum. Immortalized myoblasts were isolated from wild-type and *Dok7*^*2YF*^ embryos and grown as described previously^29^.

HEK 293 cells were purchased from and authenticated by ATCC (ATCC Cat CRL-1573). Cells were grown at 37 °C in the same medium as described above for C2C12 myoblasts and transfected using Lipofectamine 3000 Transfection Reagent Kit (Thermofisher Scientific). All cell lines tested negative for mycoplasma contamination using the e-Myco Plus PCR detection kit.

### Antibody treatment of C2 myotubes

Three days after C2C12 myotubes had formed, the cultures were treated for 30 min with 10 nM biotinylated Fabs in complex with 2.5 nM streptavidin, 10 nM IgGs, or 0.5 nM recombinant neural AGRIN-B8 (R&D Systems). Myotubes were homogenized at 4 °C in lysis buffer (50 mM sodium chloride, 30 mM triethanolamine, pH 7.5, 50 mM sodium fluoride, 5 mM EDTA, 5 mM EGTA, 2 mM sodium orthovanadate, 1 mM *N*-ethylmaleimide, 1 mM sodium tetrathionate, 10 μM pepstatin, plus complete protease inhibitor mix (Roche)). NP-40 was added to a final concentration of 1%, and the extract was incubated with rocking for 30 min at 4 °C. Insoluble proteins were removed by centrifugation at 12,000 rpm for 20 min at 4 °C. The supernatant was precleared for 1 h at 4 °C with Protein G-agarose beads (Sigma-Aldrich) before incubation overnight at 4 °C with antibodies against MUSK (MUSK 1A)^30^. Complexes were incubated for 4 h with Protein G-agarose beads. The beads were subsequently washed (three times for 9 min) in lysis buffer containing 1% NP-40. Proteins were eluted from the beads with 1% SDS in lysis buffer.

### Isolation of MUSK and DOK7 from muscle

Whole leg muscles or cultured muscle cells were homogenized at 4 °C in lysis buffer (50 mM sodium chloride, 30 mM triethanolamine pH 7.5, 50 mM sodium fluoride, 5 mM EDTA, 5 mM EGTA, 2 mM sodium orthovanadate, 1 mM *N*-ethylmaleimide, 1 mM sodium tetrathionate, 10 μM pepstatin, plus complete protease inhibitor mix (Roche)). NP-40 was added to a final concentration of 1%, and the extract was incubated with rocking for 30 min at 4 °C. Insoluble proteins were removed by centrifugation at 12,000 rpm for 20 min at 4 °C. The supernatant was pre-cleared for 1 h at 4 °C with Protein G-agarose beads (Sigma-Aldrich) before overnight incubation at 4 °C with antibodies against MUSK (MUSK 1A)^30^ or goat anti-DOK7 (R&D Systems, AF 6398), followed by incubation for 4 h with Protein G-agarose beads. The beads were subsequently washed (three times for 9 min) in lysis buffer containing 1% NP-40. Proteins were eluted from the beads with 1% SDS in lysis buffer.

### Western blotting

Proteins were fractionated by SDS–PAGE and transferred to PVDF membranes. Blots were probed with antibodies against MUSK (R&D Systems, AF562), phosphotyrosine (Millipore, 05-321) or DOK7 (1916), as described previously^18–20,24^. Antibodies against CRK (BD Bioscience, 610035) and CRK-L (Santa Cruz Biotechnology, sc-365092) have been described previously^20^. We quantified the band intensities with a ChemiDoc imaging system (BioRad), as described previously^24^. The graphs show the mean values from at least three separate experiments. A two-sided Student’s *t*-test was used to determine statistical significance and was conducted using GraphPad Prism 9.0 software.

### Whole-mount muscle immunohistochemistry

Diaphragm muscles were dissected from E18.5 embryos and postnatal mice in oxygenated L-15 medium. The muscles were pinned onto Sylgard-coated dissection dishes, fixed for 1.5 h in 1% PFA and blocked for 1 h in PBS with 3% BSA (Sigma IgG free) and 0.5% Triton X-100 (PBT). Diaphragm muscles were stained with Alexa 488-conjugated anti-BGT (Invitrogen) to label AChRs and with antibodies against neurofilament-L (Synaptic Systems, 171002), β-TUBIII (Synaptic Systems 302302) or synapsin 1/2 (Synaptic Systems, 106002) to label motor axons and nerve terminals, respectively^31^. The antibodies were force-pipetted into the muscle, and the muscles were incubated overnight at 4 °C on an orbital shaker in a humidified chamber. Diaphragm muscles were washed 10 times over the course of 5 h with PT (PBS with Triton X-100) at room temperature and rinsed in PBS before the muscle was whole-mounted in 50% glycerol. Muscles from at least three mice of each genotype were analysed for each experiment. Images were acquired with a Zeiss LSM 800 confocal microscope using ZEN software. Adjustments to detector gain and laser intensity were made to avoid saturation. The number and size of synapses, the density of synaptic AChRs, the width of the endplate zone, the extent of denervation and the co-localization index (synapsin/AChRs) were quantified using FIJI/ImageJ software, as described previously^32^. A two-sided Student’s *t*-test was used to determine statistical significance and was conducted using GraphPad Prism 9.0 software.

### Staining single muscle fibres

Tibialis anterior muscles were dissected in oxygenated L-15 medium, pinned to a Sylgard-coated dish and fixed in 2% PFA (in PBS) for 2 h. After several rinses in PBS, one to three myofibres were manually teased with fine forceps. Fixed myofibres were blocked for 2 h at room temperature in PBS containing 5% BSA, 1% normal goat serum, and 0.04% saponin. Fibres were then incubated with primary antibodies overnight at 4 °C, washed three times for 5 min with PBS containing 0.04% saponin, incubated with secondary antibodies for 2 h at room temperature, washed again, and mounted in VectaShield (Vector Laboratories). Antibodies against CRK-L (Santa Cruz Biotechnology, sc-365092) were used and the postsynaptic membrane was visualized by staining with Alexa Fluor 488–anti-BGT (Invitrogen).

### Cryosection immunohistochemistry

Limb muscles were embedded in optimal cutting temperature (OCT) medium and frozen on a dry-ice platform. Ten-micrometre sections, collected onto poly-l-lysine-coated glass slides, were fixed in 1–4% PFA for 10 min, washed in PBS with 3% BSA (PB) three times for 5 min, permeabilized with PB + 0.5% X-Triton (PBT) for 10 min, washed in PB and incubated overnight at 4 °C with primary antibodies against CRK-L (Santa Cruz Biotechnology, sc-365092) in PBT in a humidified chamber. Sections were washed in PB three times for 5 min before overnight incubation at 4 °C with secondary antibodies and Alexa Fluor 488–anti-BGT (Invitrogen), diluted in PBS, in a humidified chamber. Sections were washed three times for 5 min in PB, then PBS, before mounting in Vectashield anti-fade mounting medium.

### Behaviour

All-limb grip strength was measured using a grip-strength apparatus (Bioseb). Mice were allowed to grip the grid with both forelimbs and hindlimbs, and the mouse was pulled back steadily, until the mouse lost grip on the grid. The grip strength meter digitally displayed the maximum force applied (in grams) as the grasp was released. The mean measurement from six consecutive trials was taken as an index of all-limb grip strength. Mice were given an interval of 10–15 s between trials. Body weight was determined after all grip-strength measurements to analyse for potential co-variability. To enhance the robustness and reliability of the grip-strength assessment, all measurements were taken by the same experimenter^33^.

Motor function of male and female mice at P60 was assessed on a rotarod (AccuRotor four-channel, Omnitech Electronics). Mice were placed on the rotarod (3.0-cm rotating cylinder) rotating at 2.5 rpm, and the speed of rotation was increased linearly to 40 rpm over the course of 5 min. The time to fall from the rod was measured. Each mouse was subjected to three trials with 5-min intervals, and we recorded the longest latency to fall from the three trials. A two-sided Student’s *t*-test was used to determine statistical significance and was conducted using GraphPad Prism 9.0 software.

### Development of synthetic antibodies

The full-length extracellular region (E22 to T494 of mouse MUSK and E22 to T495 of human MUSK), including the Fz domain and the C-terminal flanking sequence (D307 to T494 of mouse MUSK and K314 to T495 of human MUSK) were expressed as a C-terminal fusion with the Avi and His_6_ tags using the secretion signal sequence of mouse IgkVIII in EXPI293 cells with the ExpiFectamine 293 Transfection kit (Thermo Fisher Scientific) using standard procedures provided by the vendor. The proteins were purified from the filtered culture supernatant using a HiTrap Nickel column (GE Healthcare) and biotinylated in vitro using the BirA enzyme in the presence of 0.5 mM biotin and 10 mM ATP. The biotinylated proteins were further purified using a Superdex S75 10/300 column (GE Healthcare).

Sorting of an antibody phage-display library was performed as described previously^34^. In brief, a phage-display library was first sorted with all four antigens at 100 nM in the first round, followed by sorting with a single antigen at 100, 50 and 20 nM in the second, third and fourth rounds, respectively. To enrich for clones that bind to both human and mouse Fz domains, we used multiple sorting strategies in which alternate antigens were used in successive rounds (for example, human Fz; mouse ECD; human ECD). Individual clones were screened using phage enzyme-linked immunosorbent assay (ELISA) with the four antigens^34^, and the DNA sequences of clones bound to all of the antigens were determined.

The Fab proteins with the Avi tag at the C terminus of the heavy chain of selected clones were produced from *Escherichia coli* and biotinylated as described previously^34^. The mouse IgG2a-LALAPG sample of clone X17 was produced using a modified version of the pFUSE-mIgG2a-Fc vector (InvivoGen) containing the LALAPG mutations in the Fc region^26^ and human CH1 domain and the pFUSE-CLIg vector (InvivoGen). This chimeric antibody consisted of a human Fab and mouse Fc sequences. In addition, we exchanged the mouse Fc sequences with those from human IgG1, containing LALA mutations, to generate hIgG1-X17, hIgG1-X2 and hIgG1-X3 antibodies.

### Affinity measurements

The affinities of antibody clones in the Fab and IgG formats were measured using a bead-binding assay^35–37^. A biotinylated human antigen protein was immobilized on Dynabeads M280 streptavidin beads (Thermo Fisher Scientific) by rapidly mixing 100 μl of tenfold diluted beads in PBSB (PBS containing 0.5% bovine serum albumin (BSA, GeminiBio)) and 100 μl of 50 nM protein. The beads were then blocked with 2 μM biotin, washed twice with PBSB and resuspended in 1 ml PBSB. This reaction was appropriately scaled for the number of measurements when necessary. Five microlitres of the diluted beads and 20 μl of an antibody sample were mixed in a well of a 96-well polypropylene plate (Greiner Bio-One, catalogue number 650261) and incubated at room temperature for 30 min with gentle shaking. Samples were transferred to the wells of a 96-well filter plate (Millipore MultiScreen HTS HV, 0.45 mm, Thermo Fisher); the liquid was removed using a vacuum manifold and the wells were washed three times with 200 μl ice-cold PBSB using the vacuum manifold. The beads were stained with anti-human Fab antibody labelled with Alexa Fluor 647 (Jackson Immuno Research, Alexa Fluor 647 AffiniPure Goat Anti-Human IgG, F(ab′)_2_ fragment specific, 109-605-097). Following washing, the beads were suspended in 70 μl PBSB and analysed using an iQue screener (Sartorius) or an Intellicyt HTFC system. The resulting titration curves were analysed by nonlinear least-squared fitting of a 1:1 binding model using the GraphPad Prizm software.

### Half-life of antibody in blood

Mice were injected intraperitoneally with antibodies. Mouse blood samples were centrifuged, and supernatants were diluted 2,000-fold in PBSB. Antibody levels were measured using the bead assay described above except that the binding reaction was performed at 4 °C. The half-life was determined by nonlinear least squares fitting of the median fluorescence intensities with a single exponential curve.

### Phosphopeptide pull-down assay

HEK 293 cells were transfected with plasmids encoding HA-tagged DOK7 and HA-tagged CRKI at 37 °C for 48h (Lipofectamine 3000, Thermofisher Scientific). After 48 h, the transfected cells were homogenized at 4 °C in lysis buffer; NP-40 was added to a final concentration of 1%, and the extract was incubated with rocking for 30 min at 4 °C. Insoluble proteins were removed by centrifugation at 12,000 rpm for 20 min at 4 °C. The supernatants were precleared for 1 h at 4 °C with streptavidin-agarose beads (Sigma-Aldrich).

Four biotinylated phosphopeptides ((1) ELLLDRLHPNPMYQRMPLLLN, (2) ELLLDRLHPNPMp(Y)QRMPLLLN, (3) ELLLDRLHPAPMp(Y)QRMPLLLN, and (4) ELLLDRLHPNPMp(Y)AAAPLLLN (Thermofisher Scientific)) were immobilized on streptavidin-agarose beads and incubated overnight at 4 °C in lysis buffer (50 mM sodium chloride, 30 mM triethanolamine, pH 7.5, 50 mM sodium fluoride, 5 mM EDTA, 5 mM EGTA, 2 mM sodium orthovanadate, 1 mM *N*-ethylmaleimide, 1 mM sodium tetrathionate, and 10 μM pepstatin, plus complete protease inhibitor mix (Roche)), containing 1% NP-40. The cell extracts, pre-cleared on streptavidin-agarose beads, were incubated overnight at 4 °C with biotinylated phosphopeptides immobilized on streptavidin-agarose beads. The beads were subsequently washed (three times for 9 min) in lysis buffer containing 1% NP-40. Proteins were eluted from the beads with 1% SDS in lysis buffer. Western blotting was performed using antibodies against HA tag (Abcam, ab49969).

### Quantitative PCR with reverse transcription (RT–qPCR)

Total RNA was isolated from muscles of E18.5 wild-type and *Dok7*^*CM*^ embryos using TRIZOL reagent (Invitrogen) and reverse transcribed with Superscript-III First strand kit (Invitrogen). Real-time qPCR was performed on a LightCycler 480 (Roche) using SYBR Green Master kit (Roche). PCRs were performed using primer pairs: 5′-CTGGTGAAAAGGACCTCTCGAAG-3′ and 5′-CCAGTTTCACTAATGACACAAACG-3′ for *Hprt*, 5′-TCAGCCTCAGAAGAGCGTGTTG-3′ and 5′-GCCTCAGAAGAGGAACTGGATAG-3′ for *Dok7*. Samples were run in triplicate and *Dok7* expression level was normalized to *Hprt* expression.

### Statistics and reproducibility

No statistical method was used to predetermine sample size. No data were excluded from the analyses. The experiments were not randomized. The investigators were not blinded to the genotype of the mice with the exception of the motor performance experiments.

### Reporting summary

Further information on research design is available in the Nature Research Reporting Summary linked to this paper.

## Online content

Any methods, additional references, Nature Research reporting summaries, source data, extended data, supplementary information, acknowledgements, peer review information; details of author contributions and competing interests; and statements of data and code availability are available at 10.1038/s41586-021-03672-3.

### Supplementary information

Supplementary Figures This file contains the uncropped western blots for Figures 1–3 and Extended Data Figures 2, 3, 4 and 8.

Reporting Summary

Video 1 MuSK agonist antibody X17 reverses impaired mobility of *Dok7*^*CM/CM*^ mice A *Dok7*^*CM/CM*^ mouse was injected with hIgG1-X17 at P4, P24 and P44. Antibody treatment was then discontinued. The video shows the movement of this *Dok7*^*CM/CM*^ mouse as well as a wildtype mouse at P100. At P100, the *Dok7*^*CM/CM*^ mouse moved infrequently and displayed motor deficits, notably dragging of his hindlimbs. Treatment with hIgG1-X17 was reinitiated at P100, and the second video was recorded 7 days later. At P107, the *Dok7*^*CM/CM*^ mouse showed improved use of the hindlimb muscles and movement throughout the cage.

### Source data

Source Data Fig. 1

Source Data Fig. 2

Source Data Fig. 3

Source Data Fig. 4

Source Data Extended Data Fig. 1

Source Data Extended Data Fig. 2

Source Data Extended Data Fig. 3

Source Data Extended Data Fig. 5

Source Data Extended Data Fig. 6

Source Data Extended Data Fig. 7

Source Data Extended Data Fig. 8

Source Data Extended Data Table 1

## Figures and Tables

**Extended Data Table 1:**
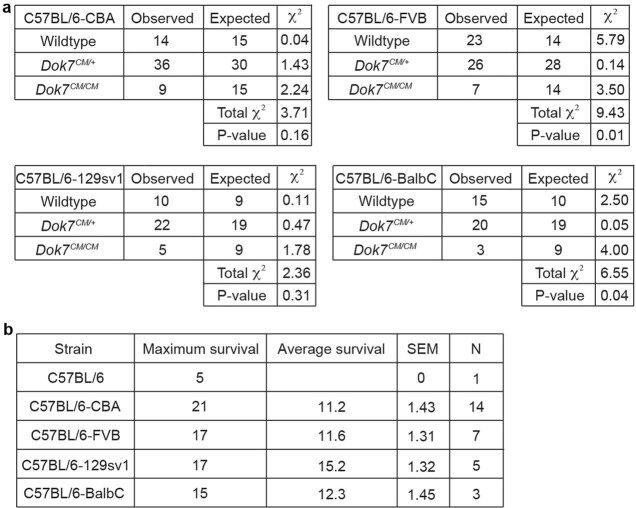
*Dok7*^*CM/CM*^ mice on a mixed genetic background survive for approximately two weeks postnatally Source data

We used a mixed genetic background of mice to analyse the survival of *Dok7*^*CM/CM*^ mice. *Dok7*^*CM/+*^ mice on a C57BL/6 background were crossed to wild-type CBA, 129sv1, FVB, or BALB/c mice. Heterozygous F1 progeny were then intercrossed to produce *Dok7*^*CM/CM*^ mice on a mixed background. We determined the genotype of the progeny at P5–P10 or post-mortem. **a**, χ^2^ analysis of F2 mice shows that the occurrence of genotypes is unlikely to occur by chance, indicating that homozygous *Dok7*^*CM/CM*^ mice in each mixed genetic background survive postnatally. **b**, The table shows the average and maximum survival time (days) of homozygous *Dok7*^*CM/CM*^ mice on a mixed genetic background. Despite surviving for up to three weeks after birth, DOK7 expression, MUSK phosphorylation and the organization of nerve terminals and AChRs were similar in E18.5 inbred C57BL/6 and mixed breed C57BL/6-CBA mice carrying the same *Dok7* mutation.

**Extended Data Table 2:**
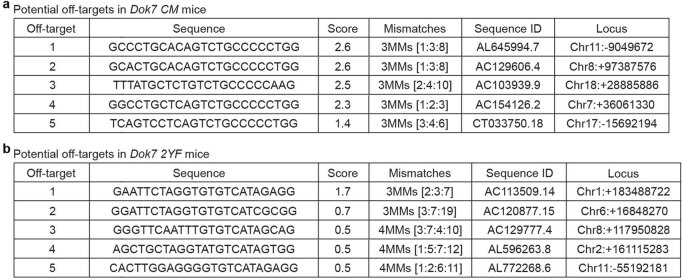
Sequence analysis of potential off-target sites failed to identify mutations in these genes

The top-ranked potential off-target genes in *Dok7*^*CM*^ and *Dok7*^*2YF*^ mice are indicated.

## Data Availability

Raw data generated from this study are available upon a reasonable request. Source data are provided with this paper.
