# Supplementary material for: Mechanism of disease and therapeutic rescue of Dok7 congenital myasthenia
Source: Nature. 2021 Jun 23;595(7867):404–8. doi: 10.1038/s41586-021-03672-3 (PMC8277574; doi:10.1038/s41586-021-03672-3)
Supplement: Supplementary file 1 — This file contains the uncropped western blots for Figures 1–3 and Extended Data Figures 2, 3, 4 and 8. [file 41586_2021_3672_MOESM1_ESM.pdf]

nature portfolio

https://doi.org/10.1038/s41586-021-03672-3

Supplementary information

Mechanism of disease and therapeutic rescue of *Dok7* congenital myasthenia

In the format provided by the authors and unedited

This file contains the uncropped blots for the figures listed below:

Figure	Page
Fig. 1	3
Fig. 2	4
Fig. 3	5
Extended data Fig. 2	6
Extended data Fig. 3	7
Extended data Fig. 4	8
Extended data Fig. 8	9

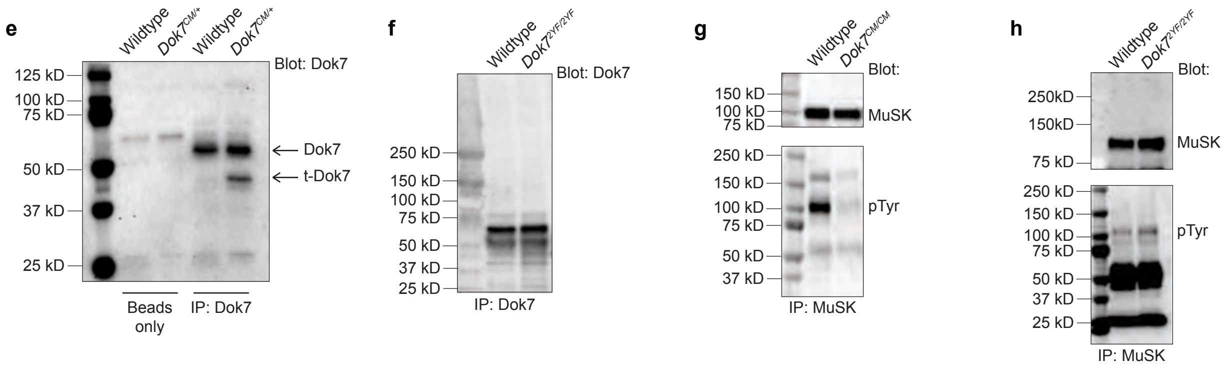
e
Wildtype
Dok7CM/+
Wildtype
Dok7CM/+
Blot: Dok7
125 kD
100 kD
75 kD
50 kD
37 kD
25 kD
Dok7
t-Dok7
Beads only
IP: Dok7
f
Wildtype
Dok72YF/2YF
Blot: Dok7
250 kD
150 kD
100 kD
75 kD
50 kD
37 kD
25 kD
IP: Dok7
g
Wildtype
Dok7CM/CM
Blot:
150 kD
100 kD
75 kD
MuSK
250 kD
150 kD
100 kD
75 kD
50 kD
37 kD
pTyr
IP: MuSK
h
Wildtype
Dok72YF/2YF
Blot:
250kD
150kD
75 kD
MuSK
250 kD
150 kD
100 kD
75 kD
50 kD
37 kD
25 kD
pTyr
IP: MuSK

b

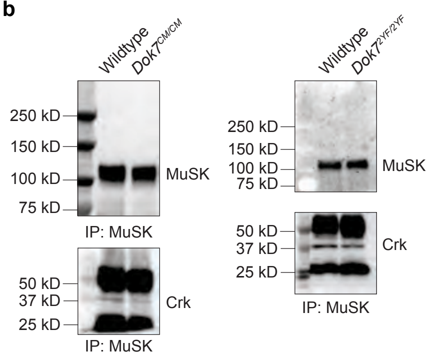
Wildtype
Dok7CM/CM
250 kD
150 kD
100 kD
75 kD
MuSK
IP: MuSK
50 kD
37 kD
25 kD
Crk
IP: MuSK
Wildtype
Dok72YF/2YF
250 kD
150 kD
100 kD
75 kD
MuSK
50 kD
37 kD
25 kD
Crk
IP: MuSK

d

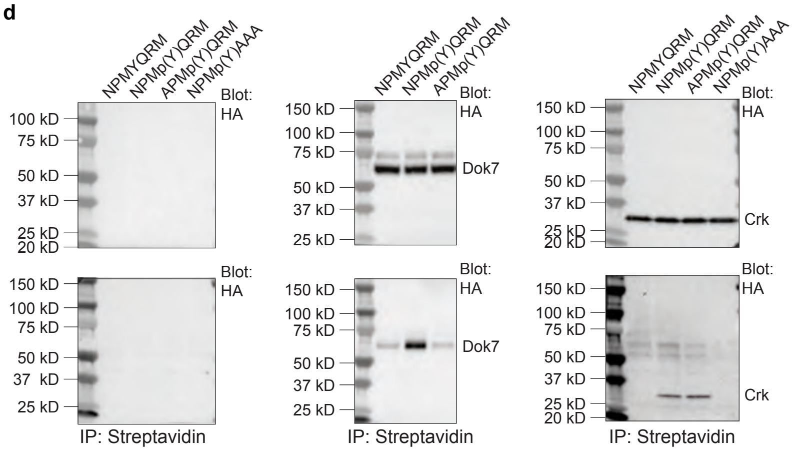
NPMYQRM
NPMp(Y)QRM
APMp(Y)QRM
NPMp(Y)AAA
Blot: HA
100 kD
75 kD
50 kD
37 kD
25 kD
20 kD
Blot: HA
150 kD
100 kD
75 kD
50 kD
37 kD
25 kD
IP: Streptavidin
NPMYQRM
NPMp(Y)QRM
APMp(Y)QRM
Blot: HA
150 kD
100 kD
75 kD
50 kD
37 kD
25 kD
Dok7
Blot: HA
150 kD
100 kD
75 kD
50 kD
37 kD
25 kD
Dok7
IP: Streptavidin
NPMYQRM
NPMp(Y)QRM
APMp(Y)QRM
NPMp(Y)AAA
Blot: HA
150 kD
100 kD
75 kD
50 kD
37 kD
25 kD
20 kD
Crk
Blot: HA
150 kD
100 kD
75 kD
50 kD
37 kD
25 kD
20 kD
Crk
IP: Streptavidin

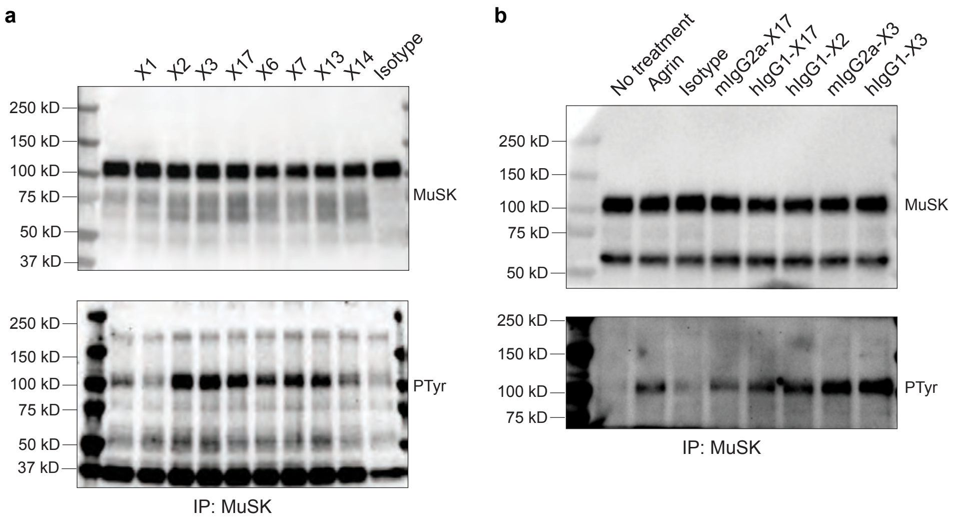
a
X1
X2
X3
X17
X6
X7
X13
X14
Isotype
250 kD
150 kD
100 kD
75 kD
50 kD
37 kD
MuSK
PTyr
IP: MuSK
b
No treatment
Agrin
Isotype
mIgG2a-X17
hIgG1-X17
hIgG1-X2
mIgG2a-X3
hIgG1-X3
250 kD
150 kD
100 kD
75 kD
50 kD
MuSK
PTyr
IP: MuSK

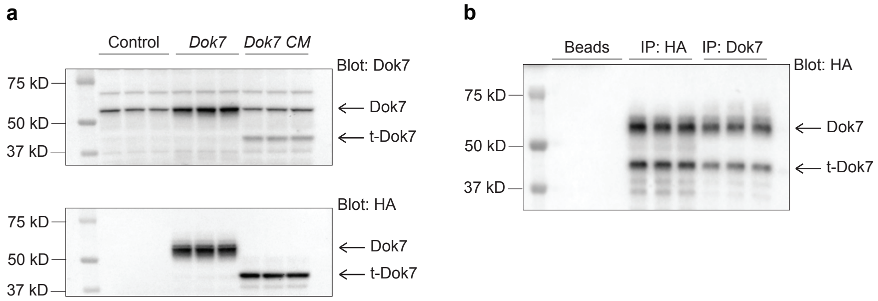
a
Control
Dok7
Dok7 CM
Blot: Dok7
75 kD
50 kD
37 kD
Dok7
t-Dok7
Blot: HA
75 kD
50 kD
37 kD
Dok7
t-Dok7
b
Beads
IP: HA
IP: Dok7
Blot: HA
75 kD
50 kD
37 kD
Dok7
t-Dok7

a

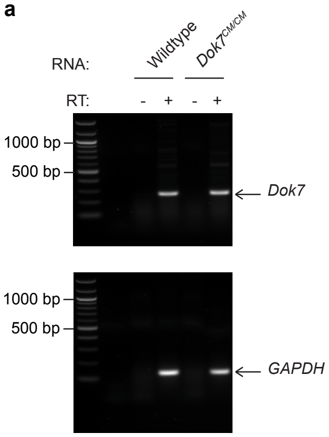

Wildtype
Dok7CM/CM
RNA:
RT:
- + - +
1000 bp
500 bp
Dok7
1000 bp
500 bp
GAPDH

c

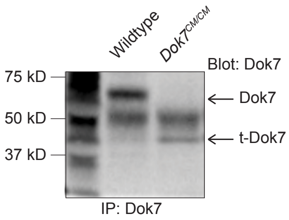

Wildtype
Dok7CM/CM
Blot: Dok7
75 kD
50 kD
37 kD
Dok7
t-Dok7
IP: Dok7

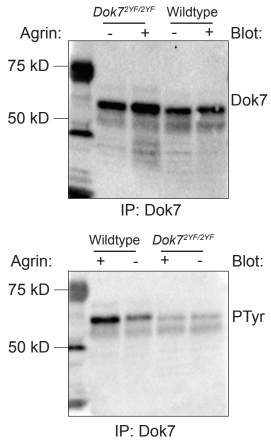
Dok7 2YF/2YF
Wildtype
Agrin:
- + - +
Blot:
75 kD
50 kD
Dok7
IP: Dok7
Wildtype
Dok7 2YF/2YF
Agrin:
+ - + -
Blot:
75 kD
50 kD
PTyr
IP: Dok7

d

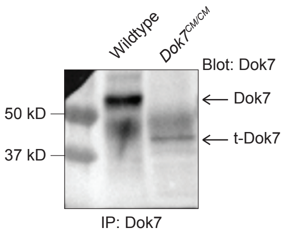

Wildtype
$Dok7^{CM/CM}$
Blot: Dok7
Dok7
t-Dok7
50 kD
37 kD
IP: Dok7

e

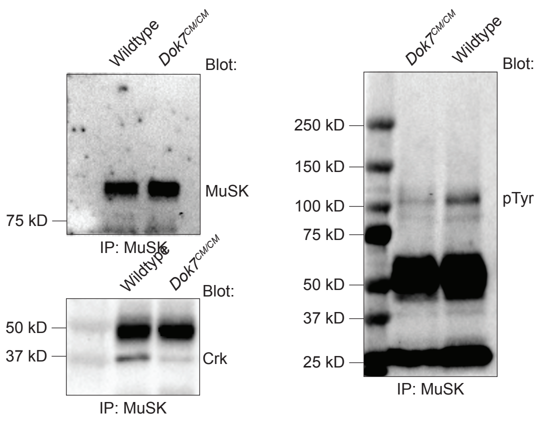

Wildtype
$Dok7^{CM/CM}$
Blot:
MuSK
75 kD
IP: MuSK
Wildtype
$Dok7^{CM/CM}$
Blot:
50 kD
37 kD
Crk
IP: MuSK
$Dok7^{CM/CM}$
Wildtype
Blot:
250 kD
150 kD
100 kD
75 kD
50 kD
37 kD
25 kD
pTyr
IP: MuSK
