# Supplementary material for: Mechanism of disease and therapeutic rescue of Dok7 congenital myasthenia
Source: Nature. 2021 Jun 23;595(7867):404–8. doi: 10.1038/s41586-021-03672-3 (PMC8277574; doi:10.1038/s41586-021-03672-3)
Supplement: Supplementary file 2 — Reporting Summary [file 41586_2021_3672_MOESM2_ESM.pdf]

nature research

Corresponding author(s): Steven J. Burden and Shohei Koide

Last updated by author(s): 5/14/2021

Reporting Summary

Nature Research wishes to improve the reproducibility of the work that we publish. This form provides structure for consistency and transparency in reporting. For further information on Nature Research policies, see our Editorial Policies and the Editorial Policy Checklist.

Statistics

For all statistical analyses, confirm that the following items are present in the figure legend, table legend, main text, or Methods section.

n/a	Confirmed	
☐	☒	The exact sample size (*n*) for each experimental group/condition, given as a discrete number and unit of measurement
☐	☒	A statement on whether measurements were taken from distinct samples or whether the same sample was measured repeatedly
☐	☒	The statistical test(s) used AND whether they are one- or two-sided<br>*Only common tests should be described solely by name; describe more complex techniques in the Methods section.*
☒	☐	A description of all covariates tested
☒	☐	A description of any assumptions or corrections, such as tests of normality and adjustment for multiple comparisons
☐	☒	A full description of the statistical parameters including central tendency (e.g. means) or other basic estimates (e.g. regression coefficient) AND variation (e.g. standard deviation) or associated estimates of uncertainty (e.g. confidence intervals)
☐	☒	For null hypothesis testing, the test statistic (e.g. *F*, *t*, *r*) with confidence intervals, effect sizes, degrees of freedom and *P* value noted<br>*Give P values as exact values whenever suitable.*
☒	☐	For Bayesian analysis, information on the choice of priors and Markov chain Monte Carlo settings
☒	☐	For hierarchical and complex designs, identification of the appropriate level for tests and full reporting of outcomes
☒	☐	Estimates of effect sizes (e.g. Cohen's *d*, Pearson's *r*), indicating how they were calculated

Our web collection on statistics for biologists contains articles on many of the points above.

Software and code

Policy information about availability of computer code

Data collection	ChemiDoc imaging system (Biorad), Zeiss ZEN Software (Zeiss Blue), Grip Strength apparatus (Bioseb), Rotarod (AccuRotor four-channel), Omnitech Electronics, Inc), LightCycler 480 (Roche), iQue screener (Sartorius). All softwares are from third party developers.
Data analysis	ImageJ v1.52P, Graphpad Prism 9.0, iQue screener (Sartorius), FlowJo V9.9.6. (BD), LightCycler 480 (Roche). All softwares are from third party developers.

For manuscripts utilizing custom algorithms or software that are central to the research but not yet described in published literature, software must be made available to editors and reviewers. We strongly encourage code deposition in a community repository (e.g. GitHub). See the Nature Research guidelines for submitting code & software for further information.

Data

Policy information about availability of data

All manuscripts must include a data availability statement. This statement should provide the following information, where applicable:

- Accession codes, unique identifiers, or web links for publicly available datasets
- A list of figures that have associated raw data
- A description of any restrictions on data availability

Raw data generated from this study are available upon a reasonable request.

Field-specific reporting

Please select the one below that is the best fit for your research. If you are not sure, read the appropriate sections before making your selection.

☒ Life sciences ☐ Behavioural & social sciences ☐ Ecological, evolutionary & environmental sciences

For a reference copy of the document with all sections, see nature.com/documents/nr-reporting-summary-flat.pdf

Life sciences study design

All studies must disclose on these points even when the disclosure is negative.

Sample size	No statistical method was used to predetermine sample size. The initial sample size for any assay was estimated from past experiments, allowing us to select a sample size that had low variance among the group and ensuring reproducibility.
Data exclusions	No data were excluded in the study.
Replication	All experiment were performed as triplicate at minimum unless otherwise stated. All attempts at replication were successful.
Randomization	The experiments were not randomized.
Blinding	The Investigators were not blinded to the genotype of the mice except for the motor performance experiments. Animals were allocated to groups according to their genotype.

Reporting for specific materials, systems and methods

We require information from authors about some types of materials, experimental systems and methods used in many studies. Here, indicate whether each material, system or method listed is relevant to your study. If you are not sure if a list item applies to your research, read the appropriate section before selecting a response.

Materials & experimental systems

n/a	Involved in the study	
☐	☒	Antibodies
☐	☒	Eukaryotic cell lines
☒	☐	Palaeontology and archaeology
☐	☒	Animals and other organisms
☒	☐	Human research participants
☒	☐	Clinical data
☒	☐	Dual use research of concern

Methods

n/a	Involved in the study	
☒	☐	ChIP-seq
☒	☐	Flow cytometry
☒	☐	MRI-based neuroimaging

Antibodies

Antibodies used

Human anti-MuSK (MuSK 1A) (gift from Dr. Kevin O'Connor). Immunoprecipitation 2ug/mg of protein lysate
Goat anti-Dok7 (R&D Systems, AF 6398). Immunoprecipitation 5ug/mg of protein lysate
Goat anti-MuSK (R&D Systems, AF562). Western Blots 1/200
Mouse anti-Phosphotyrosine (Millipore, 05-321). Western Blots 1/1000
Rabbit anti-Dok7 (Homemade, #1916). Western Blots 1/400
Mouse anti-Crk (BD Bioscience, 610035). Western Blots 1/1000
Mouse anti-CrkL (Santa Cruz Biotechnology, sc-365092). Immunofluorescence 1/100
Rabbit anti-NeurofilamentL (Synaptic Systems, 171002). Immunofluorescence 1/3000
Rabbit anti-BetaIIITubulin (Synaptic Systems, 302302). Immunofluorescence 1/3000
Rabbit anti-Synapsin 1/2 (Synaptic Systems, 160002). Immunofluorescence 1/2000
Mouse anti-HA tag (ab49969, Abcam). Western Blots 1/5000
Bungarotoxin, Alexa-488 conjugate (Thermofisher Scientific, B13422). Immunofluorescence 1/1000
Alexa Fluor 647 AffiniPure Goat anti-Human IgG, Fab fragment specific (Jackson ImmunoResearch, 109-605-097). Immunofluorescence 1/200

Validation

Human anti-MuSK is an antibody for the detection of MuSK protein. Validation statement and citation is provided with Takata K, et al. Characterization of pathogenic monoclonal autoantibodies derived from muscle-specific kinase myasthenia gravis patients. JCI Insight. 2019;4(12):e127167. Published 2019 Jun 20. doi:10.1172/jci.insight.127167.
Goat anti-Dok7 (R&D Systems, AF 6398) is a polyclonal antibody for the detection of Human, mouse, and rat Dok7 in immunoprecipitation and western blots. Citations are provided with 1) Sarcoglycan Alpha Mitigates Neuromuscular Junction Decline in Aged Mice by Stabilizing LRP4 (Authors: K Zhao, C Shen, L Li, H Wu, G Xing, Z Dong, H Jing, W Chen, H Zhang, Z Tan, J Pan, L Xiong, H

Wang, W Cui, XD Sun, S Li, X Huang, WC Xiong, L Mei. J. Neurosci., 2018;0(0). Species: Mouse. Sample Types: Cell Lysates. Applications: Western Blot), and 2) Sorbs1 and -2 Interact with CrkL and Are Required for Acetylcholine Receptor Cluster Formation (Authors: Hallock P, Chin S, Blais S, Neubert T, Glass D. Mol Cell Biol, 2015;36(2):262-70. Species: Mouse. Sample Types: Cell Lysates).
Goat anti-MuSK (R&D Systems, AF562) is a polyclonal antibody for the detection of mouse and rat MuSK in ELISAs and western blots. Citations are provided with 1) Characterization of pathogenic monoclonal autoantibodies derived from muscle-specific kinase myasthenia gravis patients (Authors: K Takata, P Stathopoul, M Cao, M Mané-Damas, ML Fichtner, ES Benotti, L Jacobson, P Waters, SR Irani, P Martinez-M, D Beeson, M Losen, A Vincent, RJ Nowak, KC O'Connor JCI Insight, 2019;4(12):. Species: Mouse. Sample Types: Cell Lysates. Applications: Immunoprecipitation), 2) IgG-specific cell-based assay detects potentially pathogenic MuSK-Abs in seronegative MG (Authors: S Huda, P Waters, M Woodhall, MI Leite, L Jacobson, A De Rosa, M Maestri, R Ricciardi, JM Heckmann, A Maniaol, A Evoli, J Cossins, D Hilton-Jon, A Vincent. Neurol Neuroimmunol Neuroinflamm, 2017;4(4):e357. Species: Human. Sample Types: Whole Cells. Applications: ICC), 3) DOK7 gene therapy enhances motor activity and life span in ALS model mice (Authors: S Miyoshi, T Tezuka, S Arimura, T Tomono, T Okada, Y Yamanashi. EMBO Mol Med, 2017;0(0):. Species: Mouse. Sample Types: Tissue Homogenates. Applications: Western Blot), 4) Laminin is Instructive and Calmodulin Dependent Kinase II is Non-Permissive for the Formation of Complex Aggregates of Acetylcholine Receptors on Myotubes in Culture (Authors: Raphael Vezina-Aud Matrix Biol, 2016;0(0):. Species: Mouse. Sample Types: Whole Cells. Applications: Neutralization), 5) MuSK myasthenia gravis IgG4 disrupts the interaction of LRP4 with MuSK but both IgG4 and IgG1-3 can disperse preformed agrin-independent AChR clusters (Authors: Koneczny, Inga, Cossins, Judith, Waters, Patrick, Beeson, David, Vincent, Angela. PLoS ONE, 2013;8(11):e80695. Species: Human. Sample Types: Cell Lysates. Applications: Immunoprecipitation.
Mouse anti-Phosphotyrosine (Millipore, 05-321) is a monoclonal antibody clone 4G10 that detects tyrosine phosphorylated proteins in all species. This antibody is validated for use in immunocytochemistry, immunohistochemistry, immunoprecipitation, and western blot. Citations are provided with 1) ATP synthase promotes germ cell differentiation independent of oxidative phosphorylation. (Authors: Teixeira, FK; Sanchez, CG; Hurd, TR; Seifert, JR; Czech, B; Preall, JB; Hannon, GJ; Lehmann, R. Nature cell biology 17 689-96 2015), 2) Hyperosmotic stress activates the expression of members of the miR-15/107 family and induces downregulation of anti-apoptotic genes in rat liver (Authors: Santosa, D; Castoldi, M; Paluschinski, M; Sommerfeld, A; Häussinger, D. Scientific reports 5 12292 2015), 3) The tyrosine phosphatase SHP-1 regulates hypoxia inducible factor-1α (HIF-1α) protein levels in endothelial cells under hypoxia (Authors: Alig, SK; Stampnik, Y; Pircher, J; Rotter, R; Gaitzsch, E; Ribeiro, A; Wörnle, M; Krötz, F; Mannell, H. PloS one 10 e0121113 2015), 4) Phospho-tyrosine dependent protein-protein interaction network (Authors: Grossmann, A; Benlasfer, N; Birth, P; Hegele, A; Wachsmuth, F; Apelt, L; Stelzl, U; Molecular systems biology 11 794 2015), 5) HSP90 inhibitor AUY922 induces cell death by disruption of the Bcr-Abl, Jak2 and HSP90 signaling network complex in leukemia cells. (Authors: Tao, W; Chakraborty, SN; Leng, X; Ma, H; Arlinghaus, RB; Genes & cancer 6 19-29 2015)
Rabbit anti-Dok7 (Homemade, #1916) is a polyclonal antibody that detects the PTB domain of Mouse Dok7 in western blots.
Mouse anti-Crk (BD Bioscience, 610035) is a polyclonal antibody for the detection of mouse Crk in western blots. Citations are provided with 1) Purification of pseudopodia from polarized cells reveals redistribution and activation of Rac through assembly of a CAS/Crk scaffold. (Authors: Cho SY, Klemke RL. J Cell Biol. 2002; 156(4):725-736), 2) Cbl-transforming variants trigger a cascade of molecular alterations that lead to epithelial mesenchymal conversion. (Authors: Fournier TM, Lamorte L, Maroun CR, et al. Mol Biol Cell. 2000; 11(10):3397-3410), 3) A direct interaction between JNK1 and CrkII is critical for Rac1-induced JNK activation. (Authors: Girardin SE, Yaniv M. EMBO J. 2001; 20(13):3437-3446), 4) APS facilitates c-Cbl tyrosine phosphorylation and GLUT4 translocation in response to insulin in 3T3-L1 adipocytes. (Authors: Liu J, Kimura A, Baumann CA, Saltiel AR. Mol Cell Biol. 2002; 22(11):3599-3609), 5) Increased C-CRK proto-oncogene expression is associated with an aggressive phenotype in lung adenocarcinomas. (Authors: Miller CT, Chen G, Gharib TG, et al. Oncogene. 2003; 22(39):7950-7957).
Mouse anti-CrkL (Santa Cruz Biotechnology, sc-365092) is a monoclonal antibody for detection of Crk-L of mouse, rat and human origin by western blot, immunoprecipitation, immunofluorescence, and ELISA. Citations are provided with 1) Crk Adaptor Proteins Regulate NK Cell Expansion and Differentiation during Mouse Cytomegalovirus Infection (Authors: Nabekura, T. et al. 2018. J. Immunol. 200: 3420-3428), 2) KSHV-TK is a tyrosine kinase that disrupts focal adhesions and induces Rho-mediated cell contraction (Authors: Gill, MB. et al. 2015. The EMBO journal. 34: 448-65), 3) A Novel Micropeptide Encoded by Y-Linked LINC00278 Links Cigarette Smoking and AR Signaling in Male Esophageal Squamous Cell Carcinoma (Authors: Wu S. et al. 2020 Cancer Res. 1;80(13):2790-2803), 4) Proteomic analysis of desmosomes reveals novel components required for epidermal integrity (Authors: Badu-Nkansah KA et al. 2020 Mol. Biol. Cell 31(11):1140-1153).
Rabbit anti-Neurofilament L (Synaptic Systems, 171002) is a polyclonal antibody for the detection of human, rat and mouse Neurofilament L in western blots, immunoprecipitation, immunocytochemistry, immunohistochemistry. Citations are provided with 1) Impaired Neurofilament Integrity and Neuronal Morphology in Different Models of Focal Cerebral Ischemia and Human Stroke Tissue. (Authors: Mages B, Aleithe S, Altmann S, Blietz A, Nitzsche B, Barthel H, Horn AKE, Hobusch C, Härtig W, Krueger M, Michalski D, et al.
Frontiers in cellular neuroscience (2018) 12: 161), 2) Conditional deletion of L1CAM in human neurons impairs both axonal and dendritic arborization and action potential generation. (Authors: Patzke C, Acuna C, Giam LR, Wernig M, Südhof TC. The Journal of experimental medicine (2016) 2134: 499-515).
Rabbit anti-BetaIIITubulin (Synaptic Systems, 302302) is a polyclonal antibody for the detection of human, rat and mouse Beta-III Tubulin in western blots, immunoprecipitation, immunocytochemistry, immunohistochemistry. Citations are provided with 1) Missense mutation of Fmr1 results in impaired AMPAR-mediated plasticity and socio-cognitive deficits in mice. (Authors: Prieto M, Folci A, Poupon G, Schiavi S, Buzzelli V, Pronot M, François U, Pousinha P, Lattuada N, Abelanet S, Castagnola S, et al. Nature communications (2021) 121: 1557), 2) The Actin Nucleator Cobl Is Critical for Centriolar Positioning, Postnatal Planar Cell Polarity Refinement, and Function of the Cochlea. (Authors: Haag N, Schüler S, Nietzsche S, Hübner CA, Strenzke N, Qualmann B, Kessels MM. Cell reports (2018) 249: 2418-2431.e6).
Rabbit anti-Synapsin 1/2 (Synaptic Systems, 16002) is a polyclonal antibody for the detection of human, rat and mouse Synapsin in western blots, immunoprecipitation, immunocytochemistry, and immunohistochemistry. Citations are provided with 1) Differential α2A- and α2C-adrenoceptor protein expression in presynaptic and postsynaptic density fractions of postmortem human prefrontal cortex. (Authors: Erdozain AM, Brocos-Mosquera I, Gabilondo AM, Meana JJ, Callado LF. Journal of psychopharmacology (Oxford, England) (2018) : 269881118798612), 2) Microtubule-associated protein 1B (MAP1B)-deficient neurons show structural presynaptic deficiencies in vitro and altered presynaptic physiology (Authors: Bodaleo FJ, Montenegro-Venegas C, Henríquez DR, Court FA, Gonzalez-Billault C. Scientific reports (2016) 6: 30069), 3) Altered postsynaptic-density-levels of caldendrin in the para-chloroamphetamine-induced serotonin syndrome but not in the rat ketamine model of psychosis (Authors: Smalla KH, Sahin J, Putzke J, Tischmeyer W, Gundelfinger ED, Kreutz MR. Neurochemical research (2009) 348: 1405-9).
Mouse anti-HA tag (ab49969, Abcam) is a monoclonal antibody for the detection of the HA sequence in ELISAs, immunofluorescence, immunoprecipitation and western blots. Citations are provided with 1) Split-miniSOG for Spatially Detecting Intracellular Protein-Protein Interactions by Correlated Light and Electron Microscopy (Authors: Boassa D et al. Cell Chem Biol 26:1407-1416.e5 (2019)), 2) Identification and characterization of putative Aeromonas spp. T3SS effectors. (Authors: Rangel LT et al. PLoS One 14:e0214035

(2019)), 3) Deubiquitinases Maintain Protein Homeostasis and Survival of Cancer Cells upon Glutathione Depletion (Authors: Harris IS et al. Cell Metab 29:1166-1181.e6 (2019)).
Bungarotoxin, Alexa-488 conjugate (Thermofisher Scientific, B13422). Fluorescent α-bungarotoxin conjugates can be used to facilitate identification of nicotinic AChRs and to localize neuromuscular junctions. Citations are provided with 1) A comparative assessment of lengthening followed by end-to-end repair and isograft repair of chronically injured peripheral nerves (Authors: Howarth HM, Orozco E, Lovering RM, Shah SB. Exp Neurol 2020; (331): 113328-113328), 2) A- and B-utrophin have different expression patterns and are differentially up-regulated in mdx muscle (Authors: Weir AP, Burton EA, Harrod G, Davies KE. J Biol Chem (2002) 277:45285-45290), 3) Acetylcholinesterase dynamics at the neuromuscular junction of live animals (Authors: Krejci E, Martinez-Pena y Valenzuela I, Ameziane R, Akaaboune M. J Biol Chem (2006) 281:10347-10354).

Eukaryotic cell lines

Policy information about cell lines

Cell line source(s)
C2C12 (ATCC Cat# CRL-1772), HEK-293 (ATCC Cat# CRL-1573), Immortalized myoblasts, methods described in Journal of Neuroscience 1 May 2001, 21 (9) 3151-3160; DOI: 10.1523/JNEUROSCI.21-09-03151.2001)

Authentication
C2C12, and HEK-293 cell lines were directly purchased from and authenticated by ATCC. Immortalized myoblasts were generated and authenticated in the Burden lab (see reference for methods, Journal of Neuroscience 1 May 2001, 21 (9) 3151-3160; DOI: 10.1523/JNEUROSCI.21-09-03151.2001)

Mycoplasma contamination
All cell lines were tested for mycoplasma contamination using the e-Myco Plus PCR detection kit, and cultured in the presence of antimycotic/antibiotic unless otherwise stated.

Commonly misidentified lines
(See ICLAC register)
No misidentified cell lines were used.

Animals and other organisms

Policy information about studies involving animals; ARRIVE guidelines recommended for reporting animal research

Laboratory animals
Mouse, strains: C57BL/6, CBA, FVB, 129sv1, Balb/C males and females from Birth to 5 months old

Wild animals
The study did not involve wild animals.

Field-collected samples
The study did not involve field-collected samples.

Ethics oversight
The study was approved by IACUC of NYU Langone under the protocol number of IA16-00080.

Note that full information on the approval of the study protocol must also be provided in the manuscript.
